# Part 1. Reduction of *S*-alkyl-thionocarbonates and related compounds in the presence of trialkylboranes/air

**DOI:** 10.1186/1860-5397-3-45

**Published:** 2007-12-12

**Authors:** Jean Boivin, Van Tai Nguyen

**Affiliations:** 1 Institut de Chimie des Substances Naturelles, C.N.R.S., Avenue de la Terrasse, 91198 Gif-sur-Yvette, France; 2 National Institute of Medicine Materials, 3B, Quang Trung, Hanoi, Vietnam

## Abstract

A new, mild, and environment friendly process for the reduction of *S*-alkyl-thionocarbonates, iodides and related compounds to the corresponding hydrocarbons at room temperature with good to excellent yields is described. This method uses a trialkylborane in excess (Et_3_B or Bu_3_B) and air.

## Background

The reduction of functional groups such as *S*-alkyl-dithiocarbonates (*S*-xanthates), iodides, *O*-alkyl-dithiocarbonates (*O*-xanthates) and related compounds to the corresponding alkanes is very important in organic synthesis, especially in natural products chemistry.[1–2] Deoxygenation (Barton-McCombie reaction) has been largely used to handle sensitive compounds such as sugars.[3] On the other hand, iodides and *S*-alkylxanthates are useful compounds that easily produce carbon radicals involved in radical chain reactions (cyclisation, intermolecular addition onto an olefin, etc) in which trapping of the final radical results in the transfer of the iodine atom or of the xanthate moiety.[4–5] To date, the most widely used reductive method to remove these functional groups that become superfluous at the end of the reaction process, is based on the Bu_3_SnH/AIBN combination that operates at 80°C or above. The main virtue of this method relies on its versatility and its efficiency. However, this procedure suffers from crippling drawbacks in terms of toxicity, cost, disposal, and tedious purification to remove tin residues. [6–10] However, we recently faced limits to the use of some of these methods for reductive removal of *S*-alkyl *O*-ethyl dithiocarbonates and we therefore proposed diethyl phosphite/DLP and H_3_PO_2_/Et_3_N/AIBN as attractive reagents for this purpose that circumvent these impediments.[11]

The core of the results reported in this paper and the following parts,[12–13] has been presented as communications at the X^th^ Symposium of the « Institut de Chimie des Substances Naturelles », 1–3 June 2005, Gif-sur-Yvette, France and at the "1^st^ German-French Congress in Organic Chemistry", Goslar, September 7^th^ – 11^th^, 2005. The recent observation made by Wood and his colleagues that *O*-alkylxanthates may be deoxygenated by the combination Et_3_B/air/H_2_O under similar conditions (published on the Web on 08/18/2005),[14] the subsequent work by Renaud and coworkers who studied the reduction of *B*-alkylcatecholboranes under very similar conditions,[15] as well as the very recent kinetic study published by Newcomb,[16] prompt us to report in detail our findings in this area as a series of three articles. In this first paper we report that trialkylboranes are useful reagents to achieve reduction of several radicophilic groups. A careful investigation of the process led us to develop a new method for the reduction of *S*-alkylxanthates, iodides and similar compounds to the corresponding hydrocarbons that requires a trialkylborane in excess and air as initiator, at 20°C, or even at lower temperature.[17–18]

## Results and discussion

**Figure 1:**
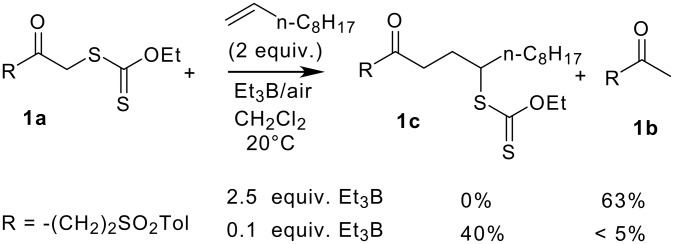
Reaction of α-acylxanthate **1a** with 1-decene and Et_3_B/air.

During the last two decades, the number of applications of Et_3_B as radical initiator has increased tremendously. As Et_3_B is known to produce radicals at 20°C, or even much lower temperature (-78°C), we thought that trialkylboranes in the presence of dioxygen would be good candidates to trigger the radical addition of α-acyl *S*-xanthates onto olefins. When testing this hypothesis, we observed that compound **1a** (Figure 1) underwent addition to decene in 40% yield when Et_3_B (0.1 equiv.)/air was used to initiate the reaction. Under these conditions, traces of alkane **1b** (< 5%) were also isolated. When 2.5 equiv. of Et_3_B were used, compound **1a** failed to yield adduct **1c** but instead was reduced to the corresponding alkane **1b** in 63% yield. No other identifiable product could be isolated. Zard and Nozaki mentioned similar reductions from α-acylxanthate[19] and α-acyliodide[20–21] respectively. These authors proposed the formation of an intermediate boron enolate that hydrolyses on work-up. A detailed report of our results concerning the addition process is given in a subsequent article.[13]

**Figure 2:**
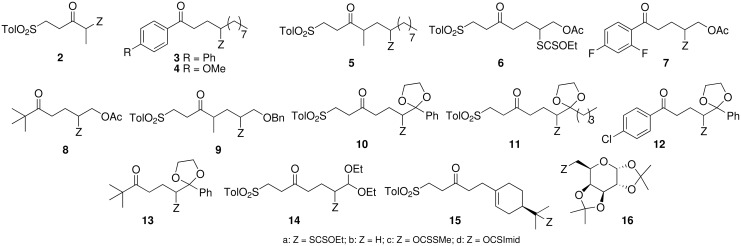
Xanthates and thionoimidazolides **2–16** and their reduced derivatives.

As the absence or the low yield of the addition product **1c** might be due to its consumption by an unanticipated reaction, we decided to examine the reactivity of *S*-alkylxanthates that do not bear a carbonyl function in the adjacent position, under similar conditions, but without added olefin. In a first set of experiments [Figure 2, Table 1, Method A, see Supporting Information File 1], we showed that the primary and secondary 2-oxoalkyl xanthates **1a** and **2a** behaved similarly (entries 1 and 2), as expected, giving high yields of compounds **1b** and **2b**, respectively. Interestingly, secondary *S*-alkylxanthates **3a–5a** were cleanly reduced to the corresponding alkanes in excellent yields (up to 80%) (Table 1, entries 3–5). When the starting material was soluble enough, there was no need for an extra solvent other than the mixture of hexanes present in the commercial Et_3_B solution (entries 3 and 6). Experimentally, in method A, a solution of xanthate, Et_3_B (5 equiv., 1M solution in hexanes), in the given solvent (if needed) was simply stirred for 2 h in the presence of air under anhydrous conditions [see Supporting Information File 1].

**Table 1:** Reduction of *S*-alkylxanthates with Et_3_B/air at 20°C according to Method A.

Entry	Xanthate	Product	Et_3_B (equiv.)	Solvent	Yield (%)

1	**1a**	**1b**	2.5	(CH_2_Cl)_2_	77
2	**2a**	**2b**	2.5	(CH_2_Cl)_2_	75
3	**3a**	**3b**	5	-	79
4	**4a**	**4b**	5	THF	79
5	**5a**	**5b**	7	Et_2_O	80
6	**7a**	**7b**	5	-	50
7	**8a**	**8b**	5	Et_2_O	63
8	**10a**	**10b**	5	(CH_2_Cl)_2_	51
9	**12a**	**12b**	5	(CH_2_Cl)_2_	45
10	**13a**	**13b**	5	Et_2_O	67
11	**14a**	**14b**	5	(CH_2_Cl)_2_	60
12	**15a**	**15b**	5	CH_2_Cl_2_	52
13	**16a**	**16b**	5	-	0 (90) ^a^

None of the reactions reported was fully optimised. For details on method A, see Experimental Section. ^a^ Yield of recovered starting material.

Secondary xanthates **7a** and **8a**, bearing an acetyl group, were also reduced, but in slightly lower yields (entries 6, 7). The reduction with Et_3_B/air is also adapted to *S*-alkylxanthates which are a part of fragile compounds such as ketals or carbonyl derivatives that contain a leaving group in the β-position. Thus, compounds **10a, 12a–14a** gave the corresponding alkanes in fair to good yields (entries 8–11). The reduction of a tertiary xanthate, without risk of any pseudo-Tchugaev thermal elimination, was also feasible in good yield as shown by reaction of compound **15a** (entry 12). Attempts to reduce a primary xanthate under these conditions failed. No trace of compound **16b** was produced when xanthate **16a** was subjected to the action of Et_3_B/air at 20°C, but, noteworthy, 90% of the starting material was recovered (entry 13).

**Scheme 1:**
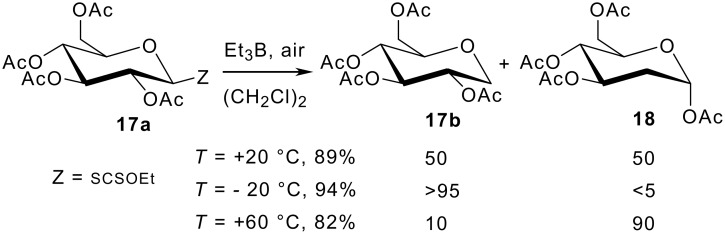
Reduction of xanthate **17a** at different temperatures with Et_3_B (5 equiv.)/air.

In a second set of experiments, we tried to gain information concerning the rate of the reduction. In this context, the Surzur-Tanner/Giese rearrangement was particularly illustrative. [22–25] The 2 → 1 migration of the acyloxy group in glycosyl radicals is well documented and allows an easy preparation of 2-deoxy-sugars. At 20°C in 1,2-dichloroethane, a 50/50 mixture of the "regular" 1-deoxy sugar **17b** and the rearranged 2-deoxy sugar **18** was produced (Scheme 1). When the temperature was raised to 60°C, the latter compound became largely predominant (ratio **17b**/**18** = 10/90). On the contrary, at low temperature (-20°C), only traces of the rearranged product (< 5%) could be detected by ^1^H NMR. Regardless of the temperature, the yield of the reduction exceeds 82%. It is important to note that, at 20°C, the rate of reduction equals the rate of the 1,2-acetyl group migration. The rate constant for the latter process has been estimated to be 4.0 x 10^2^ s^-1^ in benzene at 75°C.[26]

**Scheme 2:**
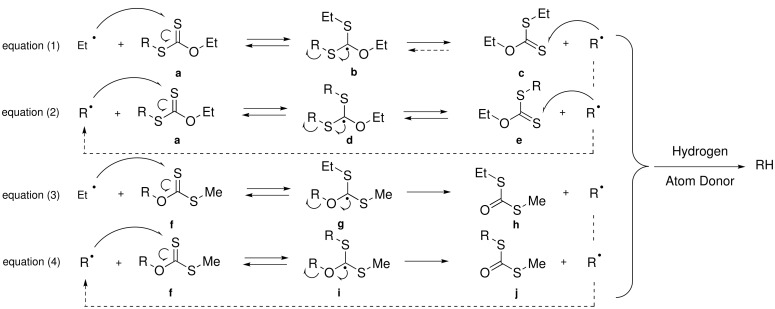
Reduction of *S*-alkylxanthates and *O*-alkylxanthates.

We then turned our attention to the reduction of *O*-alkylxanthates using the same technique (Method A). Despite our efforts, changing the solvent, the amount of Et_3_B (from 1 to 7 equiv.) or the concentrations (0.1 or 0.2 M), compound **19** led repeatedly to complex mixtures. Our interpretation is based on the following observations: – the attack of a carbon radical on the sulfur atom of a thiocarbonyl function is known to be a fast process; – the hydrogen atom transfer in the system Et_3_B/air is a relatively slow process as demonstrated above. In the case of *S*-alkylxanthates **a**, the production of radical R^•^ results from the reaction of xanthate **a** with Et^•^ radical generated from O_2_ and Et_3_B, as depicted by equation (1) (Scheme 2). When R^•^ is a secondary radical, the reaction is pulled to the right side, i.e. to the formation of the secondary radical, more stable than the primary Et^•^ . If the hydrogen atom transfer is slow, the radical may easily react with **a** or **e** [equation (2)], thus establishing a degenerate process that does not consume the starting material **a**, preserves the radical character and allows slow hydrogen atom transfer to occur. For *O*-alkylxanthates, the situation is different. The reaction of Et^•^ with **f** gives rise to **h** and R^•^ [equation (3)]. If the hydrogen atom transfer is slow, the latter reacts with **f** to afford the rearranged *S*-alkyl *S*-methyldithiocarbonate **j**. As R^•^ radical cannot react with the carbonyl function of **j**, the starting material is progressively consumed by the unwanted formation of **j** [equation (4)]. Furthermore, as the reaction proceeds, the concentration of **f** decreases, giving radical R^•^ the opportunity to undergo uncontrolled reactions.

This rationale permitted reduction of *O*-alkylxanthates. To minimise the unwanted reactions, both the concentration of R^•^ and the concentration of the substrate must be kept low. The former may be adjusted by controlling the amount of oxygen in the medium while the latter may be regulated either by high dilution technique or by slow addition of the substrate. Simply carrying out the reduction of xanthate **19** with Et_3_B (5 equiv.), under high dilution conditions (C = 0.04 M) combined with a slow addition of air with a syringe pump (40 mL/h for 2 h), led to a gratifying 27% yield of reduced material **20** (Table 2, entry 2). Increasing the amounts of Et_3_B to 10 equiv. or decreasing to 3 equiv. did not significantly improve the outcome of the reaction (Table 2, entries 3 and 4). When both air and xanthate were added slowly with two syringe pumps, the yield of reduced compound **20** was increased (Table 2, entries 6–8). The best result (57%) was obtained by the association of a highly diluted medium and slow additions of air and substrate [see Supporting Information File 1, Method B and Table 2, entry 7].

**Table 2:** Reduction of *O*-alkylxanthate 19 to 20 under various conditions.

Entry	Solvent	Concentration (M)^a,b^	Et_3_B (equiv.)	Rate of addition of xanthate **19**	Rate of addition of air	Yield (%) **20**

1	Various^c^	0.1 or 0.2	1 to 7	All at once	Open to air	<5
2	CH_2_Cl_2_	0.04	5	All at once	80 mL/2 h	27
3	(CH_2_Cl)_2_	0.02	10	All at once	80 mL/1.2 h	26
5	(CH_2_Cl)_2_	0.027	3	All at once	60 mL/0.8 h	31
6	(CH_2_Cl)_2_	0.03	10	30 min.	40 mL/2 h	44
7	(CH_2_Cl)_2_	0.02	5	15 min.	15 mL/1.5 h	57
8	(CH_2_Cl)_2_	0.12	5	15 min	15 mL/1.5 h	32

^a^ Experiments carried out on 0.5 mmol of xanthate **19**. ^b^ Concentration (M) refers to the overall amount of substrate added. ^c^ CH_2_Cl_2_, (CH_2_Cl)_2_, THF, Et_2_O

**Scheme 3:**
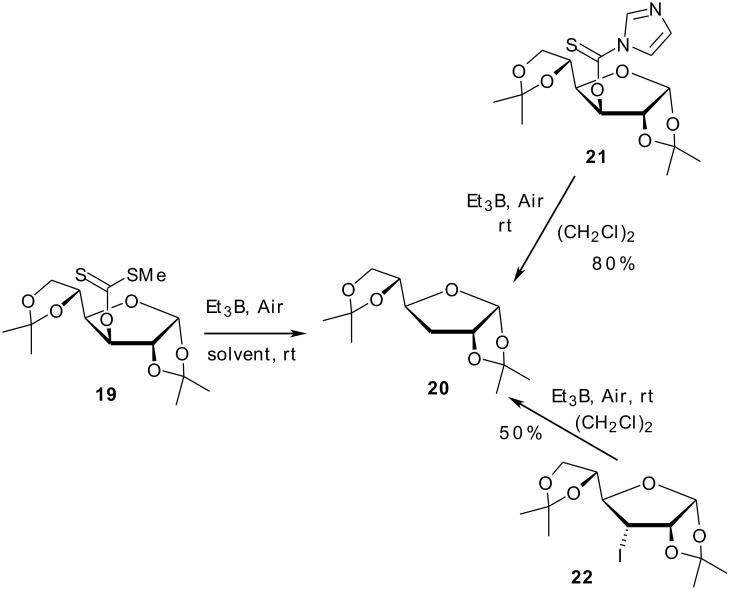
Reduction of *O*-alkyl-*S*-methyl xanthate **19**, thionoimidazolide **21** and iodide **22** by Et_3_B/air at 20°C.

Thionoimidazolide **21** and iodide **22** were also converted to the corresponding 3-deoxy glucofuranose **20**, according to method B, in 50 and 80% yields respectively (Scheme 3). The reduction of compound **21** according to method A gave a complex mixture mostly constituted of the undesired rearranged *S*-alkylimidazolide. Not unexpectedly, primary *O*-alkylxanthate **16c** and thionoimidazolide **16d**, under the same conditions, failed to produce the corresponding 6-deoxy compound but led to complex mixtures. Wood and coworkers also noticed that the deoxygenation of primary *O*-alkylxanthates under similar conditions is impractical (yield 3%, determined by GC).[14]

The comparison of entries 1 and 7 in Table 2 shows that an impressive improvement was obtained in the deoxygenation of *O*-alkylxanthates by a better understanding of the various steps involved and by subsequent modifications of the experimental procedure.

We therefore returned our attention to the reduction of *S*-alkylxanthates that sometimes led to poor or irreproducible results when Method A was employed. In particular, some dimerisation of the radical generated from the xanthate was sometimes observed by mass spectrometry. This is clearly an indication that the concentration of radicals in the medium is too high. Utilisation of Method B permitted high and reproducible yields of the corresponding alkanes to be obtained by suppressing most of the side reactions (Table 3). For example, the yield of the reduction of xanthate **8a** increased from 63% (Method A, Table 1, entry 7) to 84% (Method B, Table 3, entry 2). Under these conditions, the amounts of Et_3_B may be lowered to 3 equiv. without altering the efficiency of the reaction (Table 3, entry 3). However, when 1.5 equiv. of Et_3_B was used, the reaction failed to reach completion (Table 3, entry 4). Bu_3_B/air is also an efficient reducing agent as exemplified by conversion of xanthate **8a** to compound **8b** in a gratifying 85% yield (Table 3, entry 5). With the noticeable exception of derivative **9a** possessing a benzyloxy group in the β-position, yields of reduced compounds always exceeded 70% (Table 3, entries 1, 2–5, and 7–9).

**Table 3:** Reduction of *S*-alkylxanthates with Et_3_B/air at 20°C according to Method B.

Entry	Xanthate	Product	Borane (equiv.)	Solvent	Yield (%)

1	**6a**	**6b**	5	CH_2_Cl_2_	71
2	**8a**	**8b**	5	(CH_2_Cl)_2_	84
3	**8a**	**8b**	3	(CH_2_Cl)_2_	83
4	**8a**	**8b**	1.5	(CH_2_Cl)_2_	55 (27) ^a^
5	**8a**	**8b**	5	CH_2_Cl_2_	85 ^b^
6	**9a**	**9b**	5	CH_2_Cl_2_	42 (10) ^a^
7	**11a**	**11b**	5	Et_2_O	77
8	**13a**	**13b**	5	Et_2_O	80
9	**15a**	**15b**	5	(CH_2_Cl)_2_	70

None of the reactions reported was fully optimised. For details on method B, see Experimental Section. ^a^ Yield of recovered starting material. ^b^ Bu_3_B was used instead of Et_3_B.

**Scheme 4:**
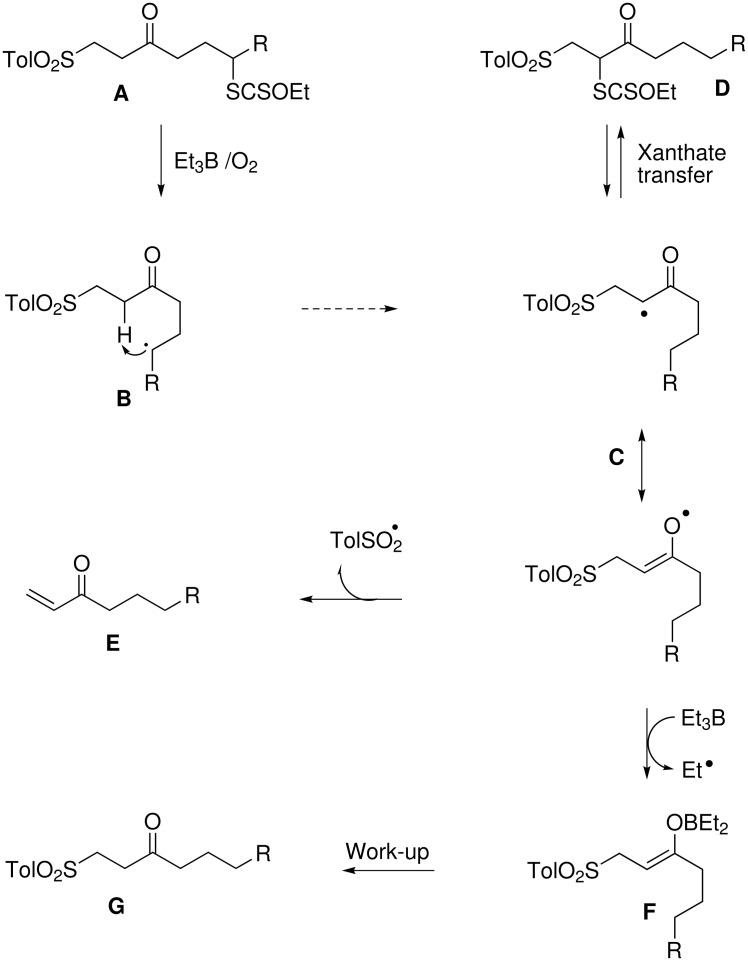
Products formed through a putative 1,5-hydrogen atom transfer.

To explain the relatively low yield of the reduction of **9a** into **9b**, the question of a putative 1,5-hydrogen atom transfer has been raised. This hypothesis calls for several remarks. Compounds **5a**, **6a**, **10a**, **11a**, and **14a** that display the same type of skeleton led to satisfactory yields of the corresponding reduced compounds (Method A or B, Table 1 and Table 3). If such a transfer occurred, radical **B** would be converted to radical **C** (represented as two mesomeric forms, Scheme 4). Radical **C** would certainly evolve rapidly to xanthate **D** or would be trapped by Et_3_B to afford a stable enolboronate **F** that would give **G** upon work-up, as postulated above for reduction of compounds **1a** and **2a**. This would thus give the same product as in the direct reduction from radical **B**, without lowering the yield of reduced compound. Alternatively, radical **C** might fragment to conjugated enone **E** and stabilised TolSO_2_^•^ radical. Neither compounds **D** nor enones **E** were ever isolated in the present work. Similarly, these compounds were not seen in a precedent work in which a "fast" hydrogen atom donor (hypophosphorus acid) or a "slow" hydrogen donor (diethylphosphite) were used on the same type of substrate.[11] Of course the hypothesis of a putative 1,5-hydrogen shift that would operate to a minor extent is not fully ruled out, and should certainly deserve further experimentation. However, one should stress that the reduction with the system Et_3_B/O_2_ is not limited to compounds where such shifts are possible and that such hydrogen migration does not pertain to the method used to generate the radical but to the intrinsic reactivity of radical **B**, as it is generally admitted that the fate of a free radical is independent of the method used to produce it. But this is beyond the scope of the present study.

## Conclusion

In this Note, we have described a new, practical and efficient method to reduce *S*-alkylxanthates, iodides, and *O*-alkylxanthates, into the corresponding alkanes under very mild conditions using commercially available reagents and simple experimental procedures [see Supporting Information File 2]. We are currently exploring the scope of the reaction by extending this process to other radicophilic species. Undeniably, the question of the origin of the hydrogen atom is compelling and an in-depth mechanistic study is needed. This intriguing point is investigated in part 2 of this series.[12]

## Supporting Information

File 1 General Procedures for Radical Reduction of Xanthates. Typical Procedures for Method A and B.

File 2 Part 1. Reduction of *S*-alkyl-thionocarbonates and related compounds in the presence of trialkylboranes/air. Detailed procedures for preparation of new compounds and their spectroscopic data.
