# Supplementary material for: Part 1. Reduction of S-alkyl-thionocarbonates and related compounds in the presence of trialkylboranes/air
Source: Beilstein J Org Chem. 2007 Dec 12;3:45. doi: 10.1186/1860-5397-3-45 (PMC2213661; doi:10.1186/1860-5397-3-45)
Supplement: File 1 — General Procedures for Radical Reduction of Xanthates. Typical Procedures for Method A and B. [file Beilstein_J_Org_Chem-03-45-s001.pdf]

General Procedures for Radical Reduction of Xanthates Part 1. Reduction of S-Alkyl-thionocarbonates and Related Compounds in the Presence of Trialkylboranes/Air

Jean Boivin,*[a] and Van Tai Nguyen*[a,b]

a) Institut de Chimie des Substances Naturelles, C.N.R.S.,

Gif-sur-Yvette 91198, France.

b) National Institute of Medicines Materials

3B, Quang Trung, Hanoi, Vietnam

jean.boivin@icsn.cnrs-gif.fr and htqt.imm@hn.vnn.vn

Method A: A solution of xanthate (0.2 mmol), Et_3B [1mL, 1M solution in hexanes (1 mmol), 5 equiv.] in the solvent (or without solvent if stated) was vigourously stirred under argon atmosphere at rt under anhydrous conditions. The argon inlet was then replaced by a drying tube filled with $CaCl_2$ open to air and stirring was continued for 2 h. The solvent was removed under reduced pressure and the residue was purified by flash chromatography.

Compound **4b**: Xanthate **4a** (0.164 g, 0.40 mmol) and Et_3B (2.0 mL, 2.0 mmol, 5 equiv.). Silica gel column chromatography (eluent heptane/EtOAc = 98/2) gave a white solid (0.092 g, 79%). m.p. 56.2-57°C; ^{1}H NMR (300 MHz, $CDCl_3$): δ=7.93 (d, ^3J=8.9 Hz, 2H), 6.92 (d, ^3J=8.9 Hz, 2H), 3.86 (s, 3H), 2.89 (t, ^3J=7.5 Hz, 2H), 1.70 (m, 2H), 1.25 (s, 16H), 0.86 (t, ^3J=6.1 Hz, 3H); ^{13}C NMR (75 MHz, $CDCl_3$): δ=199.2, 163.3, 130.3, 113.7, 55.4, 38.3, 31.9, 29.6, 29.5, 29.4, 29.3, 24.7, 22.6, 14.1; IR (neat): ν =2912, 2846, 1677, 1603, 1579, 1508, 1463, 1253, 1239, 1176, 1031, 970, 840, 809, 777, 729, 719 cm^{-1}; HRMS (ESI): $[M+Na]^+$ Calcd. for $C_{19}H_{30}O_2Na$: 313.2144. Found: 313.2134.

Method B: A solution of xanthate (0.2 mmol) in the solvent (1 mL) was added during 15 min. by a syringe pump to a vigorously stirred solution of Et_3B [1mL, 1M solution in hexanes (1 mmol)] and solvent (2 mL, ca. 0.05 M) (or without solvent if stated), under argon atmosphere at rt. Air (20 mL) was added at the same time during 2 h with a second syringe pump. The solvent was then removed under reduced pressure and the residue was purified by flash chromatography.

Compound **11b**: Et_3B (1M solution in hexanes, 18.8 mL, 18.8 mmol, 5 equiv.) in Et_2O (20 mL). Xanthate **11a** (1.89 g, 3.76 mmol) dissolved in a mixture of CH_2Cl_2 (5 mL) and Et_2O (5 mL). Silica gel column chromatography (eluent heptane/EtOAc = 9/1 then 8/2) gave **11b** (white solid, 1.062 g, 74%). M.p. 51.5-52.5 °C. ^{1}H NMR (300 MHz, $CDCl_3$):

δ=7.77 (d, ^3J=8.3 Hz, 2H), 7.36 (d, ^3J=8.3 Hz, 2H), 3.90 (brs, 4H), 3.35 (t, ^3J=7.5 Hz, 2H), 2.87 (t, ^3J=7.5 Hz, 2H), 2.45 (m, 5H), 1.59 (m, 6H), 1.29 (m, 4H), 0.89 (t, ^3J=7.1 Hz, 3H); ^{13}C NMR (75 MHz, $CDCl_3$): δ=206.0, 145.0, 136.0, 130.0, 128.0, 111.4, 64.9, 50.6, 42.7, 36.8, 36.0, 35.0, 26.0, 23.0, 22.7, 21.6, 18.0, 14.0; IR (neat): ν =2953, 1715, 1314, 1146, 1085, 815 cm^{-1}; HRMS (ESI): $[M+Na]^+$ Calcd for $C_{20}H_{30}O_5SNa$: 405.1712; Found: 405.1703.
