# Supplementary material for: Part 1. Reduction of S-alkyl-thionocarbonates and related compounds in the presence of trialkylboranes/air
Source: Beilstein J Org Chem. 2007 Dec 12;3:45. doi: 10.1186/1860-5397-3-45 (PMC2213661; doi:10.1186/1860-5397-3-45)
Supplement: File 2 — Part 1. Reduction of S-alkyl-thionocarbonates and related compounds in the presence of trialkylboranes/air. Detailed procedures for preparation of new compounds and their spectroscopic data. [file Beilstein_J_Org_Chem-03-45-s002.pdf]

Supporting Information Available

Part 1. Reduction of *S*-Alkyl-thionocarbonates and Related Compounds in the Presence of Trialkylboranes/Air

Jean Boivin,[a] and Van Tai Nguyen*[a,b]*

a) Institut de Chimie des Substances Naturelles, C.N.R.S.,

Gif-sur-Yvette 91198, France.

b) National Institute of Medicines Materials

3B, Quang Trung, Hanoi, Vietnam

jean.boivin@icsn.cnrs-gif.fr *and* htqt.imm@hn.vnn.vn

Table of Contents

**General Experimental Methods**	**Page S3**
**Reagents and Starting Materials**	**Page S3**
**Preparation of Starting Substrates**	**Page S4**
**General Procedure for Radical Additions to Olefins**	**Page S6**
**Radical Reduction of Xanthates**	**Page S20**
**Surzur-Tanner/Giese Rearrangement**	**Page S29**
**References**	**Page S31**

General Experimental Methods:

Solvents and reagents were purified according to standard literature techniques[1] and stored under argon. Experiments that required an inert atmosphere were carried out under dry argon in a flame dried glass system. Column chromatography was carried out on silica gel (230-400 mesh) by gradual elution with mixtures of n-heptane and ethyl acetate. TLC was carried out on using F_{254} SiO_2 coated aluminum plates (0,2 μm, analytical). Vizualisation was accomplished with UV light (254 nm), typical TLC indicating solution (*p*-anisaldehyde/sulfuric acid/ethanol mixture). ^1H and ^{13}C NMR spectra were recorded in deuteriochloroform on 300, 400 or 500 MHz spectrometers. The chemical shifts are reported δ unit, parts per million (ppm) relative to deuterated solvents (^1H, δ 7.269 ppm; ^{13}C, δ 77.23 ppm) or Me_4Si. Splitting pattern of an apparent multiplet associated with an averaged coupling constant were designated as s (singlet), d (doublet), t (triplet), q (quartet), m (multiplet), dd (doublet of doublet), dt (doublet of triplet), dq (doublet of quartet) and br (broadened). Coupling constants (*J* value) were reported in Hz unit. IR spectra were recorded on a FT-IR as a thin film deposit on NaCl plates or neat. ESI and high-resolution mass spectra (HRMS) were obtained with a LCT spectrometer. Deuterium incorporations were determined by analysis of isotope patterns obtained from electrospray ionization (ESI) spectra. Previously reported reaction products were identified by spectral comparison.

Reagents and Starting Materials:

Triethylborane (1M solution in hexanes) and tri-*n*-butylborane (1M in THF) were purchased from commercial suppliers, used without further purification, and stored under nitrogen atmosphere. All other commercially reagents were used as received.

Preparation of Starting Substrates:

Compounds **A**, **B**, **C**, **D**, **E**, **F**, **1a** and **2a** were prepared according to literature.[2, 3, 4]

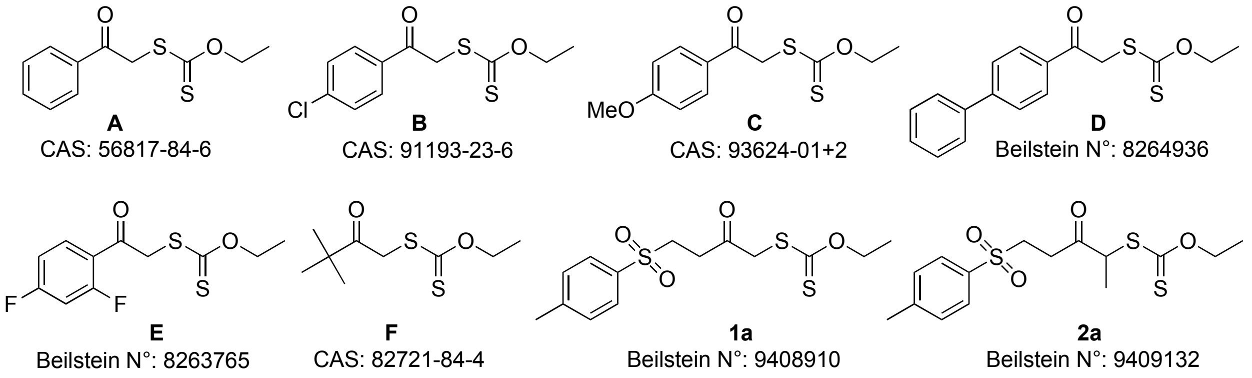

S-2-(Biphenyl-4-yl)-2-oxoethyl O-ethyl carbonodithioate (D)

^{1}H NMR (300 MHz, $CDCl_3$): δ=8.13-8.01 (m, 2H), 7.75-7.63 (m, 4H), 7.52-7.42 (m, 3H), 4.70 (s, 2H), 4.66 (q, ^3J =7.1 Hz, 2H), 1.42 (t, ^3J=7.1 Hz, 3H); ^{13}C NMR (75 MHz, $CDCl_3$): δ=213.1, 192.0, 146.6, 139.8, 134.7, 129.2, 128.6, 127.6, 127.4, 70.8, 43.7, 13.9.

S-2-(2,4-Difluorophenyl)-2-oxoethyl O-ethyl carbonodithioate (E)

^{1}H NMR (300 MHz, $CDCl_3$): δ=7.95 (dt, ^3J=6.5 Hz, $^4J_{H,F}$=8.6 Hz, 1H), 7.05-6.89 (m, 2H), 4.63 (q, ^3J=7.1 Hz, 2H), 4.57 (d, $^5J_{H,F}$=3.1 Hz, 2H), 1.41 (t, ^3J=7.1 Hz, 3H); ^{13}C NMR (75 MHz, $CDCl_3$): δ=213.0, 189.1, 133.1, 112.76, 112.48, 105.2, 104.8, 104.5, 70.7, 46.83, 13.7.

2-Butyl-2-vinyl-1, 3-dioxolane

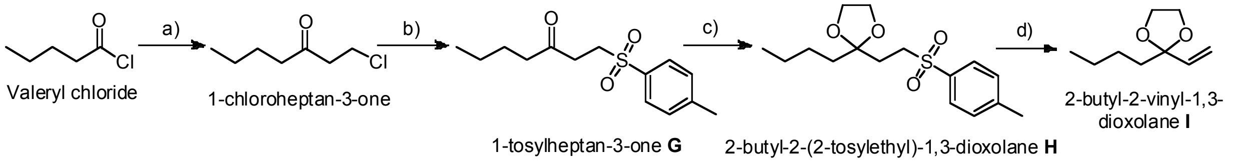

a) To a suspension of finely powdered $AlCl_3$ (30.0 g, 0.225 M) in CH_2Cl_2 (300 mL), valeryl chloride (24.12 g, 0.20 M) was added. A dry argon flow was passed through this solution to eliminate traces of HCl. The reaction mixture was then cooled at -10°C using a dry ice/ethanol bath and vigorously stirred. An ethylene stream was bubbled during approximately 3 h, until the absorption of ethylene has ceased. The temperature of the reaction is carefully maintained between -10 and -5°C. At the end of the reaction, excess of ethylene was purged out with an argon stream. The mixture was then poured on a mixture of hydrochloric acid/ice. The organic layer was separated, washed with iced water, and then with a cold aqueous solution of $NaHCO_3$ to eliminate any trace of valeric acid. After drying on Na_2SO_4, the organic layer was evaporated under reduced pressure to give 32.1 g crude of 1-chloroheptan-3-one.

b) The crude products from the Friedel-Crafts reaction was dissolved in absolute ethanol (1 L), sodium *p*-toluene sulfinate (5% H_2O) (43.2 g, 1.1 eq. vs valeryl chloride) was then added. The mixture was heated at reflux during 4 h then filtered when hot. The ethanol was evaporated in vacuo, CH_2Cl_2 was then added and the organic layer was washed, then dried on Na_2SO_4 and evaporated. The product was crystallized in ethyl acetate to give 28.2 g of 1-tosylheptan-3-one, **G**.

m.p.= 42.9-43.8 °C ^{1}H NMR (500 MHz, $CDCl_3$): δ= 7.75 (m, 2H), 7.33 (m, 2H), 3.33 (t, ^3J=7.5 Hz, 2H), 2.86 (t, ^3J =7.5 Hz, 2H), 2.43 (s, 3H), 2.39 (t, ^3J =7.0 Hz, 2H), 1.50 (quint, ^3J =7.5 Hz, 2H), 1.25 (sextuplet, ^3J =8.0 Hz, 2H), 0.86 (t, ^3J=7.5 Hz, 3H). ^{13}C NMR (75 MHz, $CDCl_3$): δ=206.5, 145.1, 136.3, 130.2, 128.2, 50.9, 42.8, 35.2, 26.0, 22.4, 21.8, 14.0; IR (neat) υ cm^{-1} 2956, 2359, 1705, 1284. Elemental analysis for $C_{14}H_{20}O_3S$: calc. C, 62.66; H, 7.51; found C, 62.72; H, 7.56.

c) To the solution of 1-tosylheptan-3-one (28.2 g, 105.1 mmol) in toluene/cyclohexane = 1/3 (220 mL). Ethylene glycol (19.57 g, 315.2 mmol, 3 eq.) and *p*-toluene-sulfonic acid (3.0 g, 15.76 mmol, 0.15 eq.) were then added. The mixture was heated at reflux with a Dean-Stark apparatus during 3 h. The organic layer was washed with an aqueous saturated solution of $NaHCO_3$ and treated as usual to give 33.5 g of crude 2-butyl-2-(2-tosylethyl)-1,3-dioxolane **H**.

m.p.= 42.9-43.5 °C; ^{1}H NMR (500 MHz, $CDCl_3$): δ=7.75 (m, 2H), 7.33 (m, 2H), 3.90-3.75 (m, 4H), 3.14 (m, 2H), 2.41 (s, 3H), 1.99 (m, 2H), 1.49 (quint, ^3J =7.4 Hz, 2H), 1.24 (sextuplet, ^3J =7.3 Hz, 4H), 0.84 (t, ^3J =7.3 Hz, 3H). ^{13}C NMR (75 MHz, $CDCl_3$): δ=144.5, 135.8, 129.8, 127.8, 109.8, 64.9, 50.4, 37.0, 29.8, 25.5, 22.0, 21.4, 13.6; IR (neat) υ cm^{-1} 2955, 2359, 1314, 1142. Elemental analysis for $C_{16}H_{24}O_4S$: calc. C, 61.51; H, 7.74 found C, 61.39; H, 7.71.

d) To a solution of *t*BuOK (32.8 g, 367.9 mmol, 3.5 eq. vs 1-tosylheptan-3-one) in dimethylsulfoxide (60 mL) at rt under argon atmosphere was added 33.5 g of crude 2-butyl-2-(2-tosylethyl)-1,3-dioxolane in dimethylsulfoxide (60 mL). The solution was stirred at rt for 48 h. The mixture was then poured into ice/water then extracted with the pentane. The organic layers were washed with water, dried on Na_2SO_4 then evaporated under reduced pressure. The crude residue was then distilled under reduced pressure to give 2-butyl-2-vinyl-1,3-dioxolane **I** (12.96 g, 41% for 4 steps). b.p. 68°C (20 mm Hg).

^{1}H NMR (300 MHz, $CDCl_3$): δ=5.73 (dd, ^3J=10.6 Hz, ^3J=17.2 Hz, 1H), 5.35 (dd, ^3J=1.9 Hz, ^3J=17.2 Hz, 1H), 5.16 (dd, ^3J=1.9 Hz, ^3J=10.6 Hz, 1H), 3.98-3.84 (m, 4H), 1.71 (brt, ^3J=7.9 Hz, 2H), 1.33 (m, 4H), 0.9 (t, ^3J=7.0 Hz, 3H); ^{13}C NMR (75 MHz, $CDCl_3$): δ=137.8, 115.2, 109.2, 64.5, 37.9, 25.5, 22.9, 14.0; IR (neat) ν =2954, 2874, 1192, 1046, 990, 932. HRMS (ESI): $[M+H]^+$ Calcd. for $C_9H_{17}O_2$: 157.1229. Found: 157.1236.

4-(Toluene-4-sulfonyl)-butan-2-one (1b) [5]

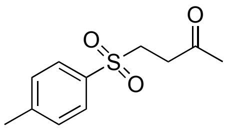

The title compound **1b** was prepared following the general procedure for radical reductions (method A, see Note), starting from xanthate **1a** (0.099 g, 0.286 mmol) and Et_3B (1M solution in hexanes, 0.7 mL, 0.7 mmol, 2.5 eq.). Silica gel column chromatography (eluent heptane/EtOAc = 9/1 then 8/2) gave a solid (0.050 g, 77%). m.p. 75.0-75.9°C; ^{1}H NMR (300 MHz, $CDCl_3$): δ=7.8 (d, ^3J=8.4 Hz, 2H), 7.4 (d, ^3J=8.1 Hz, 2H), 3.3 (t, ^3J=7.5 Hz, 2H), 2.9 (t, ^3J=7.5 Hz, 2H), 2.44 (s, 3H), 2.16 (s, 3H); ^{13}C NMR (75 MHz, $CDCl_3$): δ=203.5, 144.6, 135.6, 129.7, 127.6, 50.2, 39.0, 35.7, 21.3; IR (neat) ν =1710, 1311, 1303, 1288, 1269, 1234, 1141, 1087, 818, 768, 740, 661 cm^{-1}; HRMS (ESI): $[M+Na]^+$ Calcd. for $C_{11}H_{14}O_3SNa$: 249.0561. Found: 249.0579

General Procedure for Radical Additions to Olefins:

A solution of xanthate (1 mmol) and olefin (1,5–2 mmol) in 1,2-dichloroethane or cyclohexane (1 mL) was heated to reflux under argon atmosphere for 15 min. Then dilauroyl peroxide was added every 2 h [5% (mol/mol) vs xanthate at the fist time, followed by 2% (mol/mol) every 2 h] until complete consumption of the starting xanthate as judged by TLC. The solvent was then removed under reduced pressure and the residue was purified by flash chromatography.

S-1-(Biphenyl-4-yl)-1-oxododecan-4-yl O-ethyl carbonodithioate (3a)

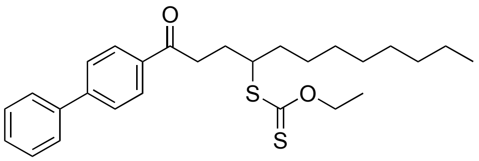

The title compound **3a** was prepared following the general procedure for the radical additions, starting from xanthate **D** (0.379 g, 1.2 mmol) and decene (0.504 g, 1.59 mmol, 3 eq.) in cyclohexane (2.4 mL) and gave a yellow solid (0.301 g, 55 % yield). The reaction was complete within 5 h (7 mol% of DLP were used).

^{1}H NMR (300 MHz, $CDCl_3$): δ=8.04 (ddd, ^3J=8.5 Hz, $^3J'$=2 Hz, and $^3J''$=1.7 Hz, 2H), 7.69 (ddd, ^3J=8.5 Hz, $^3J'$=2 Hz, and $^3J''$=1.7 Hz, 2H), 7.66-7.61 (m, 2H), 7.51-7.38 (m, 3H), 4.65 (dq, ^3J=10.7 Hz, and $^3J'$=7.1 Hz, 1H), 4.59 (dq, ^3J=11.7 Hz, and $^3J'$= 7.1 Hz, 1H), 3.86 (m, 1H), 3.18 (m, 2H), 2.29 (m, 1H), 2.02 (m, 1H), 1.75 (m, 2H), 1.49 (m, 2H), 1.40 (t, ^3J = 7.1 Hz, 3H), 1.29 (br, 10H), 0.89 (t, ^3J=6.8 Hz, 3H); ^{13}C NMR (75 MHz, $CDCl_3$): δ=214,5, 199.0, 145.8, 139.9, 135.5, 129.0, 128.7, 128.2, 127.3, 127.2, 69.7, 51.2, 35.9, 34.8, 31.9, 29.47, 29.45, 29.25, 28.7, 26.9, 22.7, 14.1, 13.8; IR (neat): ν =2918, 2850, 1673, 1604, 1207, 1054, 756 cm^{-1}; HRMS (ESI): $[M+Na]^+$ Calcd. for $C_{27}H_{36}O_2S_2Na$: 479.2054. Found: 479.2065.

O-Ethyl S-1-(4-methoxyphenyl)-1-oxododecan-4-yl carbonodithioate (4a)

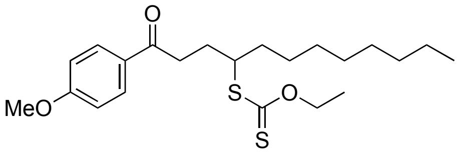

The title compound **4a** was prepared following the general procedure for the radical additions, starting from xanthate **C** (1.597 g, 5.91 mmol) and decene (1.657 g, 11.81 mmol, 2 eq.) in cyclohexane (10 mL) and gave the title compound as a yellow oil (1.513 g, 62%). The reaction was complete within 8 h (11 mol% of DLP were used).

^{1}H NMR (300 MHz, $CDCl_3$): δ=7.93 (d, ^3J=8.9, 2H), 6.91 (d, ^3J=8.9, 2H), 4.59 (m, 2H), 3.86 (s, 3H), 3.8 (m, 1H), 3.07 (m, 2H), 2.22 (m, 1H), 1.95 (m, 1H), 1.7 (q, ^3J=7.4 Hz, 2H), 1.52-1.41 (m, 2H), 1.37 (t, ^3J=7.1 Hz, 3H), 1.26 (s, 12H), 0.87 (t, ^3J=6.7 Hz, 3H); ^{13}C NMR (75 MHz, $CDCl_3$): δ=214.6, 198.0, 163.5, 130.3, 129.9, 113.7, 69.8, 55.5, 51.2, 35.5, 34.8, 31.8, 29.5, 29.4, 29.2, 28.8, 26.9, 22.7, 14.0, 13.7; IR (neat): ν =2923, 1677, 1598, 1256, 1207, 1167, 1109, 1045, 837 cm^{-1}; HRMS (ESI): $[M+Na]^+$ Calcd. for $C_{22}H_{34}O_3S_2Na$: 433.1847. Found: 433.1804.

O-Ethyl S-4-methyl-3-oxo-1-tosyltetradecan-6-yl carbonodithioate (5a) [4]

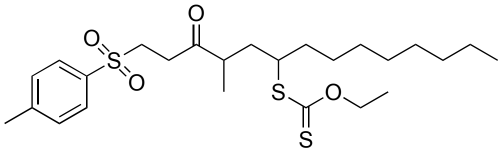

The title compound was prepared following the general procedure for the radical additions, starting from xanthate **2a** (5.0 g, 13.87 mmol) and decene (3.89 g, 27.74 mmol, 2 eq.) and gave a yellow oil (6.27 g, 90% yield). The reaction was complete within 8 h (11 mol% of DLP was used).

^{1}H NMR (300 MHz, $CDCl_3$): δ=7.76 (d, ^3J=8.0 Hz, 2H), 7.34 (d, ^3J=8.0 Hz, 2H), 4.62 (q, ^3J=7.2 Hz, 2H), 4.57 (dq ^3J=1,3 Hz, ^3J=7,17 Hz, 1H), 3.57 and 3.70 (2m, 1H), 3.33 (m, 2H), 2.64-3.05 (m, 2H), 2.43 (s, 3H), 2.12 (ddd, ^3J=4,7 Hz, $^3J'$=9.2 Hz, ^2J=14,1 Hz, 1H), 1.91 (ddd, ^3J=5,2 Hz, $^3J'$=10.1 Hz, ^2J=15,2 Hz, 1H), 1.61 (m, 2H), 1.40 (2d, ^3J=7.4 Hz, 3H), 1.30 (m, 12H), 1.09 (t, ^3J=6.5 Hz, 3H), 0.85

(t, ^3J=7.2 Hz, 3H); ^{13}C NMR (75 MHz, $CDCl_3$): δ=214.2, 209.0, 144.7, 136.1, 129.9, 127.9, 69.9, 50.5, 49.6, 44.2, 43.7, 35.1, 34.1, 33.4, 31.7, 29.1, 26.5, 22.5, 21.5, 17.5, 14.0, 13.7; IR (film): ν =1050, 1088, 1112, 1150, 1216, 1317, 1458, 1715, 2855, 2927, 2954 cm^{-1}; MS (ESI) m/z: 523.2 [M+Na]$^+$.

2-(Ethoxycarbonothioylthio)-5-oxo-7-tosylheptyl acetate (6a)

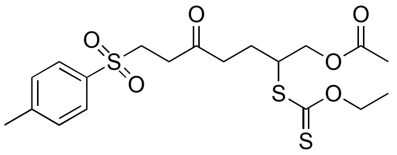

The title compound **6a** was prepared following the general procedure for the radical additions, starting from xanthate **1a** (0.349 g, 1 mmol) and allyl acetate (0.202 g, 2 mmol, 2 eq.) in 1,2-dichloroethane (1.5 mL) and gave a colourless syrup (0.350 g, 78% yield). The reaction was complete within 6 h (9 mol% of DLP was used).

^{1}H NMR (300 MHz, $CDCl_3$): δ=7.76 (d, ^3J=8.3 Hz, 2H), 7.36 (d, ^3J=8.5 Hz, 2H), 4.63 (q, ^3J=7.1 Hz, 2H), 4.27 (dd, ^3J=5 Hz and $^3J'$=11.4 Hz, 1H), 4.19 (dd, ^3J=6.1 Hz and ^3J=11.4 Hz, 1H), 3.92 (m, 1H), 3.35 (t, ^3J=7.5 Hz, 2H), 2.86 (t, ^3J=7.5 Hz, 2H), 2.63 (t, ^3J=7.5 Hz, 2H), 2.45 (s, 3H), 2.10 (m, 1H), 2.05 (s, 3H), 1.81 (m, 1H), 1.41 (t, ^3J=7.1 Hz, 2H); ^{13}C NMR (75 MHz, $CDCl_3$): δ=212.9, 204.8, 170.7, 145.0, 135.9, 130.0, 128.0, 70.5, 65.6, 50.6, 48.9, 39.7, 35.2, 24.4, 21.7, 20.8, 13.7; IR (film): ν =1737, 1715, 1379, 1362, 1313, 1216, 1144, 1109, 1085, 1040, 813, 762, 722 cm^{-1}; HRMS (ESI): [M+Na]$^+$ Calcd. for $C_{19}H_{26}O_6S_3Na$ 469.0789. Found 469.0789.

5-(2,4-Difluorophenyl)-2-(ethoxycarbonothioylthio)-5-oxopentyl acetate (7a)

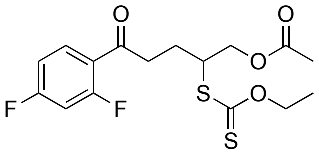

The title compound **7a** was prepared following the general procedure for the radical additions, starting from xanthate **E** (1.052 g, 3.81 mmol) and allyl acetate (0.762 g, 7.61 mmol, 2 eq.) in cyclohexane (15 mL) and gave a yellow oil (0.957 g 81% yield). The reaction was complete within 10 h (11 mol% of DLP was used).

^{1}H NMR (300 MHz, $CDCl_3$): δ=7.92 (m, 1H), 6.90 (m, 2H), 4.62 (q, ^{3}J=7.1, 2H), 4.30 (m, 2H), 4.05 (m, 1H), 3.13 (m, 2H), 2.28 (m, 1H), 2.06 (s, 3H), 2.02 (m, 1H), 1.40 (t, ^{3}J=7.1 Hz, 3H); ^{13}C NMR (75 MHz, $CDCl_3$): δ=212.9, 195.4, 170.7, 132.7, 112.4, 112.1, 105.1, 104.8, 104.4, 70.3, 65.7, 48.8, 40.5, 24.9, 20.8, 13.7; IR (neat): ν =2927, 2849, 1702, 1698, 1442, 1180 cm^{-1}. HRMS (ESI): $[M+Na]^+$ Calcd. for $C_{16}H_{18}F_2O_4S_2Na$: 399.0512. Found: 399.0510.

2-(Ethoxycarbonothioylthio)-6,6-dimethyl-5-oxoheptyl acetate (8a)

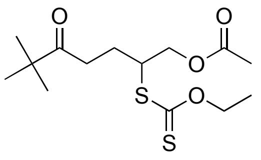

The title compound **8a** was prepared following the general procedure for the radical additions, starting from xanthate **C** (2.24 g, 10.17 mmol, 1 eq.) and allyl acetate (2.04 g, 20.34 mmol, 2 eq.) in cyclohexane (15 mL) and gave a yellow oil (2.549 g, 78% yield). The reaction was complete within 6 h (10 mol% of DLP was used).

^{1}H NMR (300 MHz, $CDCl_3$): δ=4.65 (q, ^{3}J=7.1 Hz, 2H), 4.31 (dd, ^{3}J=5 Hz, ^{3}J=11.4 Hz, 1H), 4.23 (dd, ^{3}J=6.2 Hz, $^{3}J'$=11.4 Hz, 1H), 3.95 (m, 1H), 2.68 (t, ^{3}J=7.5 Hz, 2H), 2,11 (m, 1H), 2.08 (s, 3H), 1.81 (m, 1H), 1.43 (t, ^{3}J=7.1 Hz, 2H), 1.14 (s, 9H); ^{13}C NMR (75 MHz, $CDCl_3$): δ=214.7, 213.0, 170.7, 70.3, 65.7, 49.1, 44.2, 33.6, 26.4, 24.73 20.8, 13.7; IR (neat): ν =2965, 1741, 1702, 1477, 1380, 1363, 1211, 1109, 1040 cm^{-1}; HRMS (ESI): $[M+Na]^+$ Calcd. for $C_{14}H_{24}O_4S_2Na$: 343.1014. Found: 343.1005.

S-1-(Benzyloxy)-4-methyl-5-oxo-7-tosylheptan-2-yl O-ethyl carbonodithioate (9a)

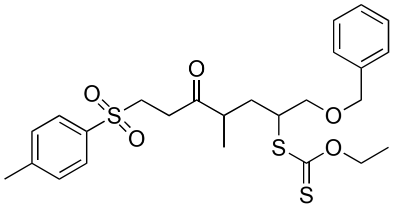

The title compound **9a** was prepared following the general procedure for the radical additions, starting from xanthate **1a** (0.360 g, 1 mmol) and allyl benzyl ether (0.296 g, 2 mmol, 2 eq.) in 1,2-dichloroethane (1.5 mL) and gave a colourless syrup (0.272 g, 54% yield). The reaction was complete within 16 h (19 mol% of DLP was used).

^{1}H NMR (300 MHz, $CDCl_3$): δ=7.78 (2d, 2H), 7.33 (m, 7H), 4.64 and 4.61 (2q, ^{3}J=7.1 Hz, 2H), 4.53 and 4.52 (2s, 2H), 3.94 and 3.88 (m, 1H), 3.68 and 3.56 (2m, 2H), 3.35 (m, 2H), 3.08-2.69 (m, 2H), 2.45 (s, 3H), 2.32 (m, 1H), 2.02 and 1.86 (2m, 1H), 1.60 (m, 1H), 1.42 and 1.40 (2t, ^{3}J=7.1 Hz, 3H), 1.12 and 1.11 (2d, ^{3}J=7.0 Hz, 3H); ^{13}C NMR (75 MHz, $CDCl_3$): 214.18, δ=209.0, 136.0, 130.0, 128.4, 128.0, 127.8, 127.7, 127.7, 127.6, 73.1, 73.0, 72.1, 72.0, 70.3, 50.6, 49.3, 48.7, 44.0, 43.7, 34.0, 33.9, 33.5, 33.4, 21.6, 17.6, 16.7, 13.8; IR (neat): ν =1712, 1596, 1453, 1313, 1212, 1146, 1109, 1086, 1040, 999, 813, 734, 696 cm^{-1}; HRMS (ESI): $[M+Na]^+$ Calcd. for $C_{25}H_{32}O_5S_3Na$: 531.1310. Found: 531.1319.

O-Ethyl S-4-oxo-1-(2-phenyl-1,3-dioxolan-2-yl)-6-tosylhexyl carbonodithioate (10a)

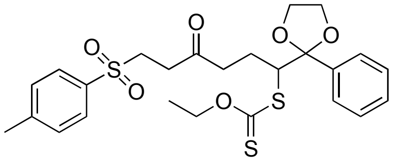

The title compound **10a** was prepared following the general procedure for the radical additions, starting from xanthate **1a** (2.43 g, 7.0 mmol) and phenyl vinyl dioxolane (2.47 g, 14.0 mmol, 2 eq.) in 1,2-dichloroethane (20 mL) and gave a white solid (2.93 g, 80% yield). The reaction was complete within 6 h (9 mol% of DLP was used).

m.p. 100.6 – 101.6°C; ^{1}H NMR (300 MHz, $CDCl_3$): δ=7.75 (d, ^{3}J=8.2 Hz, 2H), 7.48 (d, ^{3}J=7.8 Hz, 2H), 7.35 (m, 5H), 4.59 (dq, ^{3}J=1.0 Hz, ^{3}J=7.1 Hz, 2H), 4.38 (dd, ^{3}J=3.7 Hz, ^{3}J=10.3, 1H), 4.15-3.97 (m, 2H), 3.88-3.71 (m, 2H) 3.29 (t, ^{3}J=7.2 Hz, 2H), 2.79 (m, 2H), 2.55 (m, 2H), 2.45 (s, 3H), 2.05 (m, 1H), 1.69 (m, 1H), 1.40 (t, ^{3}J=7.1 Hz, 3H); ^{13}C NMR (75 MHz, $CDCl_3$): δ=205.1, 144.9, 139.9, 135.9, 130.0, 128.5, 128.1, 128.0, 126.3, 110.3, 70.4, 65.5, 65.2, 58.8, 50.6, 39.9, 35.2, 23.8, 21.6, 13.8; IR (Nujol): ν =1048; 1111; 1144; 1223; 1298; 1376; 1461; 1721; 2854; 2924; 2954 cm^{-1}; MS (ESI): *m/z* (%): 545.1 (100) $[M+Na]^+$, 561.1 (20) $[M+K]^+$.

S-1-(2-Butyl-1,3-dioxolan-2-yl)-4-oxo-6-tosylhexyl O-ethyl carbonodithioate (11a)

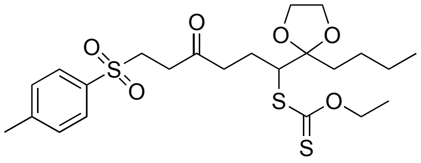

The title compound **11a** was prepared following the general procedure for the radical additions, starting from xanthate **1a** (2.0 g, 5.77 mmol) and 2-butyl-2-vinyl-1,3-dioxolane (1.35 g, 8.66 mmol, 1.5 eq) in cyclohexane (6 mL) and gave the title compound as a yellow oil (2.514 g, 87% yield). The reaction was complete after 16 h (19 mol% of DLP were used).

^{1}H NMR (300 MHz, $CDCl_3$): δ=7.78 (d, ^{3}J=8.1 Hz, 2H), 7.37 (d, ^{3}J=8.1 Hz, 2H), 4.64 (q, ^{3}J=7.1 Hz, 2H), 4.13-3.91 (m, 6H), 3.37 (ddd, ^{3}J=8.5 Hz, $^{3}J'$=6.8 Hz, $^{3}J''$=1.8 Hz, 2H), 2.86 (ddd, ^{3}J=3.8 Hz, $^{3}J'$=7.5 Hz, $^{3}J''$=11.8 Hz, 2H), 2.63 (t, ^{3}J=7.3 Hz, 2H), 2.46 (s, 3H), 2.27 (2 dt, ^{3}J=3.4 Hz, $^{3}J'$=7.7 Hz, 1H), 1.71 (m, 3H), 1.43 (t, ^{3}J=7.1 Hz, 3H), 1.33 (m, 4H), 0.90 (t, ^{3}J=7.1 Hz, 3H); ^{13}C NMR (75 MHz, $CDCl_3$): δ=215.1, 205.3, 144.9, 135.9, 130.0, 128.0, 111.9, 70.6, 66.1, 65.8, 57.3, 50.6, 39.9, 35.3, 34.9, 25.0, 23.9, 22.8, 21.6, 14.1, 13.8; IR (neat): ν =2954, 1715, 1314, 1212, 1144, 1042, 728 cm^{-1}; HRMS (ESI): $[M+Na]^+$ Calcd. for $C_{23}H_{34}O_6S_3Na$: 525.1415. Found: 525.1410

S-4-(4-Chlorophenyl)-4-oxo-1-(2-phenyl-1,3-dioxolan-2-yl)butyl O-ethyl carbonodithioate (12a)

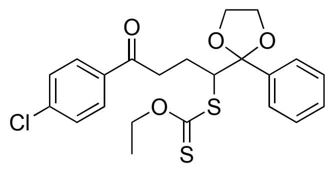

The title compound **12a** was prepared following the general procedure for the radical additions, starting from xanthate **B** (0.252 g, 0.917 mmol) and phenyl vinyl dioxolane (0.250 g, 1.421 mmol, 1.55 eq.) in 1,2-dichloroethane (1 mL) and gave the title compound as a colourless syrup (0.320 g, 77% yield). The reaction was complete within 8 h (11 mol% of DLP were used).

^{1}H NMR (300 MHz, $CDCl_3$): δ=7.80 (dt, ^{3}J=1.8 Hz, ^{3}J=8.6 Hz, 2H), 7.54-7.48 (m, 2H), 7.41-7.29 (m, 5H), 4.54 (m, 3H), 4.10 (m, 2H), 3.82 (m, 2H), 3.16-2.95 (m, 2H), 2.23 (m, 1H), 1.87 (m, 1H), 1.36 (t, ^{3}J=7.1 Hz, 3H); ^{13}C NMR (75 MHz, $CDCl_3$): δ=214.5, 198.0, 140.1, 139.3, 135.2, 129.4, 128.8, 128.5, 128.2, 126.4, 110.4, 70.3, 65.6, 65.3, 58.9, 35.8, 24.5, 13.7; IR (neat): ν =1686, 1682, 1587, 1208, 1042, 699 cm^{-1}; HRMS (ESI): [M+Na]$^+$ Calcd. for $C_{22}H_{23}{}^{35}ClO_4S_2Na$: 473.0624. Found: 473.0620 (100%) and $C_{22}H_{23}{}^{37}ClO_4S_2Na$: 475.0595. Found: 475.0595 (8.38%).

S-5,5-Dimethyl-4-oxo-1-(2-phenyl-1,3-dioxolan-2-yl)hexyl O-ethyl carbonodithioate (13a)

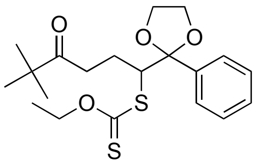

The title compound **13a** was prepared following the general procedure for the radical additions, starting from xanthate **C** (1 g, 4.538 mmol) and phenyl vinyl dioxolane (1.2 g, 6.807 mmol, 1.5 eq.) in 1,2-dichloroethane (15 mL) and gave the title compound as a yellow oil (0.957 g, 53%). The reaction was complete within 4 h (7 mol% vs xanthate of DLP was used).

^{1}H NMR (300 MHz, $CDCl_3$): δ=7.52 (dd, ^{3}J=1.5 Hz and $^{3}J'$=7.8 Hz, 2H), 7.35 (3H, m), 4.59 (2H, m), 4.43 (dd, ^{3}J=3.7 Hz and $^{3}J'$=10.4 Hz, 1H), 4.09 (m, 2H), 3.82 (m, 2H), 2.60 (m, 2H), 2.07 (m, 1H), 1.67 (m, 1H), 1.41 (t, ^{3}J=7.0 Hz, 3H), 1.07 (s, 9H); ^{13}C NMR (75 MHz, $CDCl_3$): δ=215.0,

214.6, 140.2, 128.4, 128.0, 126.4, 110.4, 70.1, 65.5, 65.2, 58.8, 44.0, 33.8, 26.5, 24.0, 13.7; IR (neat): ν =2964, 1702, 1476, 1446, 1364, 1208, 1109, 1043, 774, 698, 640 cm^{-1}; HRMS (ESI): $[M+Na]^+$ Calcd. for $C_{20}H_{28}O_4S_2Na$: 419.1327. Found: 419.1332.

S-1,1-Diethoxy-5-oxo-7-tosylheptan-2-yl O-ethyl carbonodithioate (14a)[4]

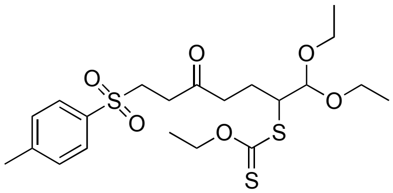

The title compound **14a** was prepared following the general procedure for the radical additions, starting from xanthate **1a** (4 g, 11.56 mmol) and olefin (8.8 mL, 7.51 g, 57.7 mmol, 5.0 eq.) in 1,2-dichloroethane (15 mL) and gave the title compound as a pale yellow oil (4.41 g, 85%). The reaction was complete within 4 h (7 mol% vs xanthate of DLP was used).

^{1}H NMR (300 MHz, CDCl3): δ=7.69 (d, ^3J=8.1 Hz, 2H,), 7.30 (d, ^3J=8.1 Hz, 2H), 4.56 (q, ^3J=7.2 Hz, 2H), 4.46 (d, ^3J=2.9 Hz, 1H), 3.96 (m, 1H), 3.58 (m, 4H), 3.32 (dt, ^3J=1.3 Hz, ^3J=7.6 Hz, 2H), 2.82 (dt, ^3J=1.5 Hz, ^3J=7.6 Hz, 2H), 2.60 (dt, ^3J=1.8 Hz, ^3J=7.3 Hz, 2H), 2.43 (s, 3H), 2.19 (m, 1H), 1.81 (m, 1H), 1.39 (t, ^3J=7.2 Hz, 3H); 1.17 (m, 6H); ^{13}C NMR (65 MHz, $CDCl_3$): δ=214.0, 204.7, 144.3, 135.5, 129.5, 127.5, 103.4, 69.8, 64.0, 63.3, 52.8, 50.1, 39.3, 34.8, 21.7, 21.1, 14.7, 13.3; IR (film): ν =2977, 2929, 2896, 1718, 1597, 1444, 1406, 1316, 1219, 1148, 1111, 1053, 815 cm^{-1}.

O-Ethyl S-2-(4-(3-oxo-5-tosylpentyl)cyclohex-3-enyl)propan-2-yl carbonodithioate (15a)

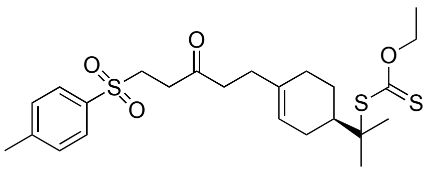

The title compound was prepared following the general procedure for the radical additions, starting from xanthate **1a** (0.510 g, 1.472 mmol) and β-pinene (0.401 g, 2.944 mmol, 2 eq.) and gave the title compound as a yellow oil (0.597 g, 83%). The reaction was complete within 2 h (5 mol% of DLP was used).

^{1}H NMR (300 MHz, $CDCl_3$): δ=7.75 (d, ^{3}J=8.2 Hz, 2H), 7.34 (d, ^{3}J=8.2 Hz, 2H), 5.30 (d, ^{3}J=4.0 Hz, 1H), 4.64 (q, ^{3}J=7.2 Hz, 2H), 3.33 (t, ^{3}J=7.5 Hz, 2H), 2.86 (t, ^{3}J=7.5 Hz, 2H), 2.50 (t, ^{3}J=7.5 Hz, 2H), 2.43 (s, 3H), 2.16 (t, ^{3}J=7.5 Hz, 2H), 2.10-1.76 (m, 7H), 1.44 (s, 6H), 1.41 (t, ^{3}J=7.2 Hz, 3H); ^{13}C NMR (75 MHz, $CDCl_3$): δ=214.2, 205.7, 144.9, 135.9, 135.7, 129.9, 127.9, 120.8, 69.3, 58.9, 50.5, 42.6, 41.0, 35.0, 30.8, 29.4, 26.9, 25.1, 24.7, 24.4, 21.6, 13.7; IR (neat): ν =1714, 1364, 1314, 1301, 1287, 1224, 1144, 1109, 1084, 1034, 813 cm^{-1}; HRMS (ESI): $[M+Na]^+$ Calcd. for $C_{24}H_{34}O_4S_3Na$: 505.1517. Found: 505.1509.

***O*-Ethyl *S*-(1,2:3,4-di-*O*-isopropylidene-α-D-galactopyranos-6-yl) dithiocarbonate (16a)**

The title compound **16a** was prepared by analogy to a procedure described in literature.[6]

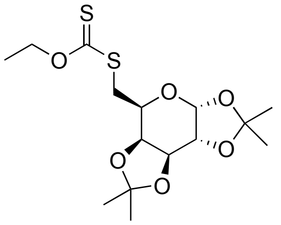

1,2,3,4-Di-*O*-isopropylidene-D-galactopyranose (0.535 g, 2.055 mmol) was dissolved in toluene (20 mL) in the presence of molecular sieves (4 A°). Tri-*n*-butylphosphine (1.027 mL, 4.111 mmol, 2.0 eq) and *O*,*O*-diisopropyl-dithiocarbonate disulfide (1.680 g, 6.929 mmol, 3.37 eq) was then added. After 20 h of stirring under reflux, water was added and the solution extracted with dichloromethane. After drying with sodium sulfate, the organic layer was concentrated in vacuo and purified by flash chromatography (heptane/EtOAc = 95/5 then 9/1) to furnish **16a** as a syrup (0.278 g, 37%).

$[\alpha]_D$ -56 (c = 0,25, CH_2Cl_2); ^{1}H NMR (300 MHz, $CDCl_3$): δ=5.55 (d, ^3J=5 Hz, 1H), 4.64 (m, 2H), 4.56 (d, ^3J=7.2 Hz, 1H), 4.49 (q, ^3J=7.2 Hz, 2H), 4.34 (dd, ^3J=2.5 Hz, ^3J=5 Hz, 1H), 4.29 (dd, ^3J=2 Hz, ^3J=7.9 Hz, 1H), 4.2 (m, 1H), 1.53 (s, 3H), 1.47 (s, 3H), 1.83 (t, ^3J=7.2 Hz, 3H), 1.35 (s, 3H), 1.33 (s, 3H); ^{13}C NMR (75 MHz, $CDCl_3$): δ=195.4, 109.7, 108.8, 96.3, 71.0, 70.9, 70.7, 70.4, 69.4, 65.4, 26.1, 26.0, 25.0, 24.5, 13.8; IR (neat): ν =2987; 1370; 1235; 1209; 1165; 1066; 999; 884 cm^{-1}; MS (ESI) m/z: 387.1 $[M+Na]^+$.

6-*O*-(Methylsulfanylthiocarbonyl)-1,2:3,4-di-*O*-isopropylidene-α-D-galactopyranose (16c)

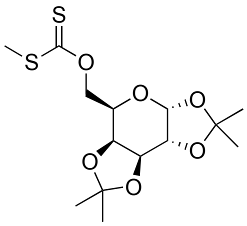

The title compound **16c** was prepared according to literature.[7]

To a solution of 1,2:3,4-di-*O*-isopropylidene-α-D-galactose (1.3 g, 5.0 mmol) in THF (25 mL) was added NaH (60% dispersion in oil, 0.33 g, 10 mmol, 2 eq.) and imidazole (10 mg). The mixture was stirred (2 h) and carbon disulfide (0.6 mL) was added. After 15 min. methyliodide (0.54 mL) was added. Acetic acid (1 mL) and dichloromethane (30 mL) were added successively. The organic layer was washed with water, then dried over Na_2SO_4 and concentrated in vacuo. The residue was purified by flash chromatography (hexane/AcOEt 9/1) to afford **16c** (1.665 g, 95%) as a syrup.

^{1}H NMR (300 MHz, $CDCl_3$): δ=5.55 (d, ^3J=5.0 Hz, 1H), 4.84 (dd, ^3J=4.7 Hz, ^3J=11.4 Hz, 1H), 4.69 (t, ^3J=7.4 Hz, 1H), 4.65 (m, 1H), 4.34 (q, ^3J=2.5 Hz, 1H), 4.29 (dd, ^3J=1.9 Hz, ^3J=7.8 Hz, 1H), 4.26-4.21 (m, 1H), 2.56 (s, 3H), 1.52 (s, 3H), 1.47 (s, 3H), 1.35 (s, 3H), 1.34 (s, 3H); ^{13}C NMR (75 MHz, $CDCl_3$): δ=215.7, 109.8, 108.9, 96.3, 72.0, 71.0, 70.7, 70.5, 65.6, 26.1, 26.0, 25.0, 24.5, 19; IR (neat): ν =1381, 1371, 1209, 1164, 1065, 1002, 887 cm^{-1}.

6-*O*-(Imidazolylthiocarbonyl)-1,2:3,4-di-*O*-isopropylidene-α-D-galactopyranose (16d)

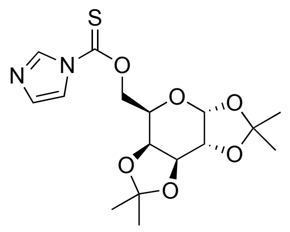

The title compound **16d** was prepared according to literature.[8,9]

1,2:3,4-di-*O*-isopropylidene-α-D-galactopyranose (0.395 g, 1.578 mmol) was dissolved in dichloromethane (10 mL) containing thiocarbonyl diimidazole (0.422g, 2.367 mmol, 1.5 eq). The reaction was allowed to reflux under an argon atmosphere for 2 h. The solvent was then removed under reduced pressure and the syrup was purified by flash chromatography using heptane/EtOAc = 7/3 and 6/4 as eluents. Compound **16d** was obtained as a syrup (0.482 g, 82%).

^{1}H NMR (300 MHz, $CDCl_3$): δ=8.17 (s, 1H), 7.46 (bs, 1H), 6.86 (bs, 1H), 5.39 (d, ^{3}J=5 Hz), 4.68 (dd, ^{3}J=3.9 Hz, ^{3}J=11.6 Hz, 1H), 4.57 (m, 1H), 4.50 (m, 1H), 4.20 (m, 1H), 4.12 (m, 2H), 1.34 (s, 3H), 1.31 (s, 3H), 1.19 (s, 3H), 1.17 (s, 3H); ^{13}C NMR (75 MHz, $CDCl_3$): δ=184.0, 136.8, 130.8, 117.9, 109.9, 108.9, 96.2, 72.0, 70.9, 70.7, 70.3, 65.5, 26.0, 25.9, 24.8, 24.4.

3,5-bis-(Acetyloxy)-2-[(acetyloxy)methyl]-6-[(ethoxycarbothioyl)-sulfanyl]tetrahydro-2H-pyran-4-yl-acetate (17a)

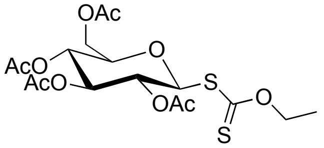

The title compound **17a** was prepared according to literature.[10]

Acetobromo-α-D-glucose (1.664 g, 4.047 mmol) was treated by potassium ethyl xanthogenate (0.973 g, 6.070 mmol, 1.5 eq.) in acetonitrile (20 mL) at room temperature under argon. After 3 h, water (30 mL) was added and the aqueous layer was extracted with CH_2Cl_2 (2x30 mL). The organic

layer was dried over $MgSO_4$ and concentrated in vacuo. The residue was purified by chromatography (heptane/AcOEt 75/25 then 70/30) to afford **17a** (1.54 g, 84%) as a syrup. The residue was crystallized from EtOH at 4°C.

m.p. 78-80°C, white solid; ^{1}H NMR (300 MHz, $CDCl_3$): δ=5.40 (d, ^3J=10.5 Hz, 1H), 5.28 (t, ^3J=9.2 Hz, 1H), 5.09 (m, 2H), 4.59 (q, ^3J=7.1 Hz, 2H), 4.19 (dd, ^3J=4.8 Hz, and $^3J'$=12.5 Hz, 1H), 4.06 (dd, ^3J=2.1 Hz, and $^3J'$=12.5 Hz, 1H), 3.77 (m, 1H), 2.01 (s, 3H), 1.97 (s, 6H), 1.95 (s, 3H), 1.37 (t, ^3J=7.1 Hz, 3H); ^{13}C NMR (75 MHz, $CDCl_3$): δ=210.0, 170.6, 170.0, 169.3, 169.3, 85.8, 76.4, 73.9, 70.7, 68.4, 68.0, 61.7, 20.7, 20.5, 13.7; IR (neat): ν =1746, 1732, 1365, 1213, 1035, 910, 729 cm^{-1}. HRMS (ESI): $[M+Na]^+$ Calcd. for $C_{17}H_{24}O_{10}S_2$.Na: 475.0709. Found: 475.0702.

α-D-1,2:5,6-Di-*O*-isopropylidene-3-*O*-[(methylthio) thiocarbonyl]-glucofuranose (19)

The title compound **19** was prepared according to literature.[11]

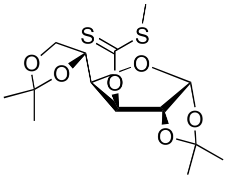

To a solution of α-D-1,2:5,6-bis-*O*-isopropylidene-glucofuranose (2.6 g, 9.99 mmol) in THF (50 mL) was added NaH (60% dispersion in oil, 0.67 g, 19.97 mmol, 2 eq) and imidazole (20 mg). The mixture was stirred under reflux for 2 h under argon atmosphere. Carbon disulfide (1.2 mL) was added. After refluxing for a further 0.5 h, methyl iodide (1.1 mL) was added. The reflux was continued for 0.5 h. After addition of acetic acid (2 mL) and dichloromethane (70 mL), the organic layer was washed with water, dried over Na_2SO_4 and concentrated in vacuo. The residue was purified by flash chromatography (hexane/AcOEt 9/1) to afford **19** (1.665 g, 95%) as a syrup.

^{1}H NMR (300 MHz, $CDCl_3$): δ=5.92 (brd 2H), 4.68, (d, ^3J=3.9 Hz, 1H), 4.32 (m, 2H), 4.11 (m, 2H), 2.60 (s, 3H), 1.55 (s, 3H), 1.42 (s, 3H), 1.33 (s, 3H), 1.32 (s, 3H); ^{13}C NMR (75 MHz, $CDCl_3$): δ

=214.8, 112.3, 109.3, 104.9, 84.1, 82.7, 79.7, 72.3, 66.9, 26.7, 26.6, 26.2, 25.2, 19.2; IR (neat): ν = 1370, 1187, 1162, 1057, 1013, 843 cm^{-1}.

α-D-1,2:5,6-bis-*O*-Isopropylidene-3-*O*-thiocarbonylimidazole-glucofuranose (21)

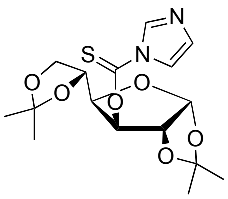

The title compound **21** was prepared according to literature.[12]

α-D-1,2:5,6-bis-*O*-isopropylidene-glucofuranose (2.058 g, 7.907 mmol) was dissolved in dichloromethane (50 mL) containing thiocarbonyl diimidazole (2.818 g, 15.8 mmol, 2 eq). The reaction was allowed to reflux under an argon atmosphere for 2 h. The solvent was then removed under reduced pressure and the dark syrup was purified by flash chromatography using heptane/EtOAc = 7/3 and 6/4 as eluents to yield quantitatively α-D-1,2:5,6-bis-*O*-isopropylidene-3-*O*-thiocarbonylimidazole-glucofuranose as a syrup.

^{1}H NMR (300 MHz, $CDCl_3$): δ=8.33 (s, 1H), 7.61 (brs, 1H), 7.07 (s, 1H), 5.95 (d, ^{3}J = 3.7 Hz, 1H), 5.84 (d, ^{3}J=2.6 Hz, 1H), 4.76 (d, ^{3}J=3.8 Hz, 1H), 4.29 (m, 2H), 4.17 (m, 1H), 4.08 (m, 1H), 1.58 (s, 3H), 1.42 (s, 3H), 1.35 (s, 3H), 1.30 (s, 3H).

3-Deoxy-3-iodo-1,2:5,6-di-O-isopropylidene- α-D-glucofuranose (22)

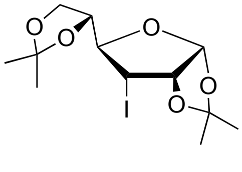

A mixture of α-D-1,2:5,6-bis-*O*-isopropylidene-glucofuranose (0.518 g, 1.99 mmol), triphenylphosphine (5.74 g, 2.19 mmol, 1.1 eq.), and imidazole (0.271 g, 3.98 mmol, 2.0 eq.), in xylene (10 mL), under argon was heated to 80°C with stirring. Iodine (0.556 g, 2.19 mmol, 1.1 eq.) was added slowly and the reaction mixture was stirred at reflux for 1.5 h. The hot mixture was poured in to a flask containing saturated aqueous $NaHSO_3$ solution (40 mL) and stirred for 10 min. Ethyl acetate (30 mL) was added, and the organic layer was washed with brine (30 mL), dried over Na_2SO_4, and concentrated. Silica gel column chromatography [elution with heptane/EtOAc (95:5 to 85/15)] afforded iodide **22** (0.169 g, 23%).[13]

^{1}H NMR (300 MHz, $CDCl_3$): δ=5.83 (d, ^3J =3.6 Hz, 1H), 4.61 (t, ^3J=4.0 Hz, 1H), 4.34 (m, 1H), 4.28 (dd, ^3J =3.9 Hz, ^3J =9.9 Hz, 1H), 4.14 (dd, ^3J =6.0 Hz, ^3J =8.5 Hz, 1H), 4.07 (dd, ^3J =6.9 Hz, ^3J =8.5 Hz, 1H), 3.78 (dd, ^3J =4.5 Hz, ^3J =9.9 Hz, 1H), 1.57 (s, 3H), 1.50 (s, 3H), 1.38 (s, 6H); ^{13}C NMR (75 MHz, $CDCl_3$): 111.7, 110.0, 103.2, 81.8, 81.5, 75.5, 65.8, 26.6, 26.6, 26.4, 25.2, 19.2; δ= IR (neat): ν = 2984, 1371, 1213, 1159, 1105, 1061, 1014, 870, 845 cm^{-1}; HRMS (ESI): $[M+Na]^+$ Calcd. for $C_{19}H_{30}O_2Na$: 393.0175. Found: 393.0177.

Radical Reduction of Xanthates:

For a description of methods A and B, see the Experimental Section in the Note.

1-Tosylpentan-3-one (2b) [14]

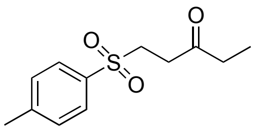

The title compound **2b** was prepared following the general procedure for the radical reductions (method A), starting from xanthate **2a** (0.150 g, 0.416 mmol) and Et_3B (1M solution in hexanes, 0.1 mL, 1.0 mmol, 2.5 eq.). Silica gel column chromatography (eluent heptane/EtOAc = 9/1 then 8/2) gave a colourless oil (0.075 g, 75%).

^{1}H NMR (300 MHz, $CDCl_3$): δ=7.76 (d, ^{3}J=8.0 Hz, 2H), 7.35 (d, ^{3}J=8.0 Hz, 2H), 3.35 (t, ^{3}J=7.5 Hz, 2H), 2.86 (t, ^{3}J=7.5 Hz, 2H), 2.47-2.39 (q, ^{3}J=7.3 Hz, 2H), 2.43 (s, 3H), 1.01 (t, ^{3}J=3Hz, 3H); ^{13}C NMR (75 MHz, $CDCl_3$): δ=206.6, 144.9, 135.9, 129.9, 127.9, 50.6, 35.9, 34.6, 21.6, 7.5; IR (neat) ν =1714, 1596, 1312, 1302, 1284, 1146, 1135, 1086, 815, 758 cm^{-1}.

1-(Biphenyl-4-yl)dodecan-1-one (3b) [15]

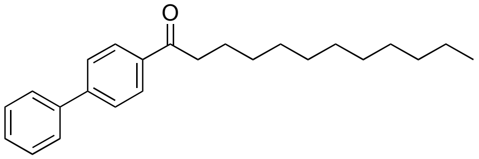

The title compound **3b** was prepared following the general procedure for the radical reductions, starting from xanthate **3a** (0.144 g, 0.32 mmol) and Et_3B (1M solution in hexanes, 1.58 mL, 1.58 mmol, 5 eq.). Silica gel column chromatography (eluent heptane/EtOAc = 98/2) gave a white solid (0.084 g, 79%).

^{1}H NMR (300 MHz, $CDCl_3$): δ=8.03 (d, ^{3}J=8.4, 2H), 7.67 (d, ^{3}J=8.3, 2H), 7.62 (d, ^{3}J=7.1, 2H), 7.44 (m, 3H), 2.98 (t, ^{3}J=7.4, 2H), 1,75 (m, 2H), 1.26 (s, 16H), 0.87 (t, ^{3}J=6.6, 3H); ^{13}C NMR (75 MHz, $CDCl_3$): δ=200.2, 145.6, 140.0, 135.8, 129.0, 128.7, 128.2, 127.3, 127.2, 38.7, 31.9, 29.6, 29.5, 29.44, 29.36, 29.2, 24.5, 22.7, 14.1; IR (neat): ν =2913, 2847, 1677, 1602, 1462, 970, 839, 758, 745, 733, 684 cm^{-1}.

4-Methyl-1-tosyltetradecan-3-one (5b)

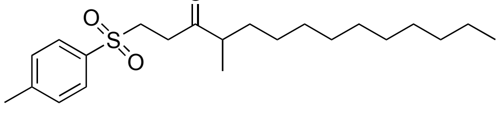

The title compound **5b** was prepared following the general procedure for the radical reductions (method A), starting from xanthate **5a** (0.188 g, 0.38 mmol) and Et_3B (1M solution in hexanes, 2.63

mL, 2.63 mmol, 7 eq.). Silica gel column chromatography (eluent heptane/EtOAc = 95/5 then 9/1) gave a white solid (0.114 g, 80%).

m.p. 50.2-51.5°C; ^{1}H NMR (300 MHz, $CDCl_3$): δ=7.79 (d, ^{3}J=8.2 Hz, 2H), 7.37 (d, ^{3}J=8.2 Hz, 2H), 3.35 (t, ^{3}J=7.6 Hz, 2H), 2.94 (m, 2H), 2.52 (m, 1H), 2.46 (s, 3H), 1.59 (m, 2H), 1.25 (brs, 16H), 1.06 (d, ^{3}J=7.0 Hz, 3H), 0.88 (t, ^{3}J=7.0 Hz, 3H); ^{13}C NMR (75 MHz, $CDCl_3$): δ=210.0, 144.8, 136.1, 129.9, 127.9, 109.9, 50.6, 46.5, 33.3, 32.8, 31.8, 29.5, 29. 3, 29.2, 27.2, 23.4, 22.6, 21.6, 16.1, 14.1; IR (neat): ν = 2912, 2847, 1708, 1596, 1464, 1408, 1312, 1305, 1258, 1140, 1085, 993, 814, 763, 693, 641 cm^{-1}; HRMS (ESI): $[M+Na]^+$ Calcd. for $C_{21}H_{34}O_3NaS$: 389.2126. Found 389.2095.

5-Oxo-7-tosylheptyl acetate (6b)

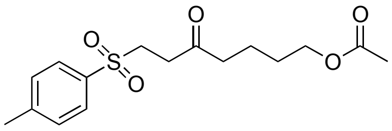

The title compound **6b** was prepared following the general procedure for the radical reductions (method B), starting from xanthate **6a** (0.67 g, 0.15 mmol) and Et_3B (1M solution in hexanes, 0.75 mL, 0.75 mmol, 5 eq.). Silica gel column chromatography (eluent heptane/EtOAc = 8/2) gave a colourless oil. Crystallization from heptane gave a white solid (0.035 g, 71%).

m.p. 45.1-46.2°C; ^{1}H NMR (300 MHz, $CDCl_3$): δ=7.79 (d, ^{3}J=8.3 Hz, 2H), 7.37 (d, ^{3}J=8.0 Hz, 2H), 4.04 (t, ^{3}J=6.0 Hz, 2H), 3.37 (t, ^{3}J=7.5 Hz, 2H), 2.89 (t, ^{3}J=7.6 Hz, 2H), 2.48 (t, ^{3}J=7.0 Hz, 2H), 2.46 (s, 3H), 2.04 (s, 3H), 1.61 (m, 4H); ^{13}C NMR (75 MHz, $CDCl_3$): δ=205.6, 171.1, 145.0, 136.0, 130.0, 128.0, 63.9, 50.6, 42.1, 35.0, 28.0, 21.6, 20.9, 20.0; IR (film): ν =1715, 1235, 1146, 1086, 1038, 816 cm$^{-1;}$ HRMS (ESI): $[M+Na]^+$ Calcd. for $C_{16}H_{22}O_5NaS$: 349.1086. Found: 349.1082.

5-(2,4-Difluorophenyl)-5-oxopentyl acetate (7b)

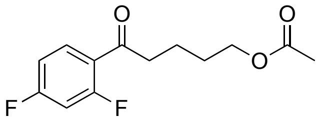

The title compound **7b** was prepared following the general procedure for the radical reductions (method A), starting from xanthate **7a** (0.252 g, 0.67 mmol) and Et_3B (1M solution in hexanes, 3.35 mL, 3.35 mmol, 5 eq.). Silica gel column chromatography (eluent heptane/EtOAc = 95/5 then 9/1) gave a white solid (0.085 g, 50%).

m.p. 49.0 – 49.9°C; white crystals; ^{1}H NMR (300 MHz, $CDCl_3$): δ=7.94 (m, 1H), 6.92 (m, 2H), 4.11 (t, ^3J=6.2 Hz, 2H), 3.00 (dt, $^5J_{H,F}$=3.3 Hz, ^3J=7.0 Hz, 2H), 2.05 (s, 3H), 1.76 (m, 4H); ^{13}C NMR (75 MHz, $CDCl_3$): δ=196.5, 171.1, 132.6, 112.4, 112.1, 104.4, 104.7, 105.1, 64.1, 42.9, 28.0, 21.0, 20.3; IR (neat): ν =1724, 1680, 1607, 1426, 1376, 1264, 1226, 1188, 1143, 1091, 1037, 988, 968, 898, 864, 829, 740, 730 cm^{-1}; HRMS (ESI): $[M+Na]^+$ Calcd. for $C_{13}H_{14}O_3F_2Na$: 279.0809. Found 279.0790.

6,6-Dimethyl-5-oxoheptyl acetate (8b)

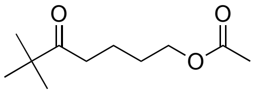

The title compound **8b** was prepared following the general procedure for the radical reductions (method A), starting from xanthate **8a** (0.215 g, 0.67 mmol) and Et_3B (1M solution in hexanes, 3.35 mL, 3.35 mmol, 5 eq.). Silica gel column chromatography (eluent: heptane/EtOAc = 9/1) gave a yellow oil (0.085 g, 63%).

Compound **8b** was also prepared by method B, starting from xanthate **8a** (0.067 g, 0.21 mmol) in $(CH_2Cl)_2$ (1 mL) and Et_3B (1.05 mL, 1.05 mmol, 5 eq.) in $(CH_2Cl)_2$ (9 mL) in 84% yield.

^{1}H NMR (300 MHz, $CDCl_3$): δ=4.02 (t, ^3J=6.2 Hz, 2H), 2.48 (t, ^3J=6.9 Hz, 2H), 2.01 (s, 3H), 1.59 (m, 4H), 1.10 (s, 9H); ^{13}C NMR (75 MHz. $CDCl_3$): δ=215.4, 171.1, 64.1, 44.0, 35.7, 28.0, 26.3,

20.9, 20.2; IR (neat): ν =2967, 1741, 1703, 1365, 1234 cm^{-1}; HRMS (ESI): $[M+Na]^+$ Calcd for $C_{11}H_{20}O_3Na$: 223.1310. Found 223.1297.

7-(Benzyloxy)-4-methyl-1-tosylheptan-3-one (9b)

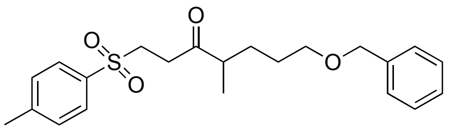

The title compound **9b** was prepared following the general procedure for the radical reductions (method B). Et_3B (1M solution in hexanes, 1.0 mL, 1.0 mmol, 5 eq.) in CH_2Cl_2 (8 mL). Xanthate **9a** (0.105 g, 0.2 mmol) dissolved in CH_2Cl_2 (1 mL). Silica gel column chromatography (eluent heptane/EtOAc = 9/1 then 8/2) gave a colourless oil (0.034 g, 42%).

^1H NMR (300 MHz, $CDCl_3$): δ=7.70 (d, ^3J=8.2 Hz, 2H), 7.24 (m, 7H), 4.40 (s, 2H), 3.35 (t, ^3J=6 Hz, 2H), 3.27 (t, ^3J=7.5 Hz, 2H), 2.85 (m, 2H), 2.47 (m, 1H), 2.37 (s, 3H), 1.70-1.27 (m, 4H), 0.99 (d, ^3J=7 Hz, 3H); ^{13}C NMR (75 MHz, $CDCl_3$): δ=209.7, 144.8, 138.2, 136.0, 129.8, 128.3, 127.9, 127.5, 72.8, 69.8, 50.5, 46.0, 33.3, 29.3, 27.2, 21.5, 16.2; IR (film): ν = 1712, 1313, 1147, 1086, 814, 734, 697 cm^{-1}; HRMS (ESI): $[M+Na]^+$ Calcd. for $C_{22}H_{28}O_4NaS$: 411.1606. Found: 411.1587.

6-(2-Phenyl-[1,3]dioxolan-2-yl)-1-(toluene-4-sulfonyl)hexan-3-one (10b)

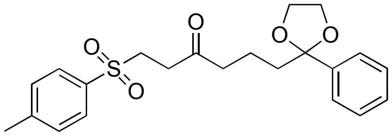

The title compound **10b** was prepared following the general procedure for the radical reductions (method A), starting from xanthate **10a** (0.2 g, 0.38 mmol) and Et_3B (1M solution in hexanes, 1.9 mL, 1.9 mmol, 5 eq.). Silica gel column chromatography (eluent heptane/EtOAc = 8/2 then 7/3) gave a white solid (0.078 g, 51%).

m.p. 120.9-121.5°C; ^{1}H NMR (300 MHz, $CDCl_3$): δ=7.76 (d, ^{3}J=8.1 Hz, 2H), 7.34 (m, 7H), 3.99 (t, ^{3}J=6.9 Hz, 2H), 3.75 (t, ^{3}J=6.9 Hz, 2H), 3.33 (t, ^{3}J=7.5 Hz, 2H), 2.84 (t, ^{3}J=7.0 Hz, 2H), 2.45 (s, 3H), 2.42 (t, ^{3}J=7.4 Hz, 2H), 1.85 (t, ^{3}J=7.7 Hz, 2H), 1.61 (m, 2H); ^{13}C NMR (75 MHz, $CDCl_3$): δ=205.9, 144.9, 142.3, 136.0, 130.0, 128.2, 128.0, 127.9, 125.6, 110.01, 64.5, 50.6, 42.5, 39.4, 35.0, 21.7, 17.8; IR (Nujol) ν =2924, 2854, 1719, 1462, 1377, 1142, 1042, 699 cm^{-1}; HRMS (ESI): $[M+Na]^+$ Calcd. for $C_{22}H_{26}O_5NaS$: 425.1399. Found 425.1378.

6-(2-Butyl-1,3-dioxolan-2-yl)-1-[(4-methylphenyl)sulfonyl]hexan-3-one (11b)

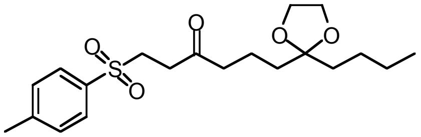

The title compound **11b** was prepared following the general procedure for the radical reductions (method B), starting from xanthate **11a** (0.108 g, 0.22 mmol) and Et_3B (1M solution in hexanes, 1.1 mL, 1.1 mmol, 5 eq.) in Et_2O (5 mL). Silica gel column chromatography with heptane/EtOAc = 9/1 then 8/2 as eluents gave a white solid (0.063 g, 77%).

^{1}H NMR (300 MHz, $CDCl_3$): δ=7.77 (d, J=8.3 Hz, 2 H), 7.36 (d, J=8.3 Hz, 2 H), 3.90 (brs, 4 H), 3.35 (t, J=7.5 Hz, 2 H), 2.87 (t, J=7.5 Hz, 2 H), 2.45 (s, t, 5 H), 1.59 (m, 6 H), 1.29 (m, 4 H), 0.89 (t, J=7.1 Hz, 3 H); ^{13}C NMR (75 MHz, $CDCl_3$): δ=206.0, 145.0, 136.0, 130.0, 128.0, 111.4, 64.9, 50.6, 42.7, 36.8, 36.0, 35.0, 26.0, 23.0, 22.7, 21.6, 18.0, 14.0; IR (neat) ν =2953, 1715, 1314, 1146, 1085, 815 cm^{-1}; HRMS (ESI): $[M+Na]^+$ Calcd. for $C_{20}H_{30}O_5SNa$: 405.1712; Found: 405.1703.

1-(4-Chlorophenyl)-4-(2-phenyl-1,3-dioxolan-2-yl)butan-1-one (12b)

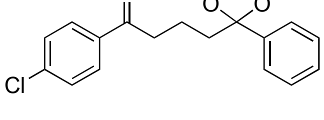

The title compound **12b** was prepared following the general procedure for the radical reductions (method A), starting from xanthate **12a** (0.1 g, 0.22 mmol) and Et_3B (1M solution in hexanes, 1.1 mL, 1.1 mmol, 5 eq.). Silica gel column chromatography with heptane/EtOAc = 98/2 as eluent gave a yellow oil (0.033 g, 45%).

^{1}H NMR (300 MHz, $CDCl_3$): δ=7.87 (d, ^3J=8.7 Hz, 2H), 7.50–7.27 (m, 7H), 4.04 (m, 2H), 3.79 (m, 2H), 2.95 (t, 7.3, 2H), 2.01 (m, 2H), 1.83 (m, 2H); ^{13}C NMR (75 MHz, $CDCl_3$): δ=198.8, 142.4, 139.3, 135.3, 129.4, 128.8, 128.28, 128.2, 128.1, 128.0, 127.9, 125.7, 110.2, 64.5, 39.7, 38.3, 18.4; IR (film): ν =1677, 1586, 1221, 1088, 752, 689 $cm^{-1;}$ HRMS (ESI): $[M+Na]^+$ Calcd. for $C_{19}H_{19}{}^{35}ClO_3Na$: 353.0920. Found: 353.0912 (75%); $[M+Na]^+$ Calcd for $C_{19}H_{19}{}^{37}ClO_3Na$: 355.0891. Found: 355.0909 (5.8%).

2,2-Dimethyl-6-(2-phenyl-1,3-dioxolan-2-yl)hexan-3-one (13b)

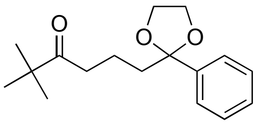

The title compound **13b** was prepared following the general procedure for the radical reductions (method B), Et_3B (1M solution in hexanes, 0.681 mL, 0.681 mmol, 5 eq.) in Et_2O (1 mL). Xanthate **13a** (0.054 g, 0.136 mmol dissolved in Et_2O (1 mL). Silica gel column chromatography (eluent heptane/EtOAc = 9/1 then 8/2) gave a yellow oil (0.076 g, 80%).

^{1}H NMR (300 MHz, $CDCl_3$): δ=7.42 (m, 2H), 7.29 (m, 3H), 4.0 (m, 2H), 3.75 (m, 2H), 2.44 (t, ^3J=7.3 Hz, 2H), 1.86 (m, 2H), 1.60 (m, 2H), 1.08 (s, 9H); ^{13}C NMR (75 MHz, $CDCl_3$): δ=215.7, 142.5, 128.1, 127.8, 125.7, 110.3, 64.5, 44.0, 39.7, 36.2, 26.4, 18.1; IR (neat): ν =2964, 2884, 1702, 1477, 1446, 1365, 1187, 1041, 1026, 946, 762, 701 cm^{-1}; HRMS (ESI): $[M+Na]^+$ Calcd. for $C_{17}H_{24}O_3Na$: 299.1623. Found 299.1634.

7.7-Diethoxy-1-(toluene-4-sulfonyl)heptan-3-one (14b)

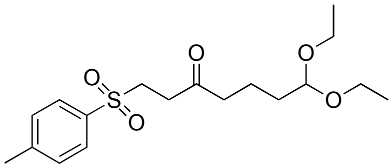

The title compound **14b** was prepared following the general procedure for the radical reductions (method B). Et_3B (1M solution in hexanes, 0.96 mL, 0.96 mmol, 5 eq.) in CH_2Cl_2 (3 mL). Xanthate **14a** (0.091 g, 0.19 mmol) dissolved in CH_2Cl_2 (1 mL). Silica gel column chromatography (eluent heptane/EtOAc = 9/1 then 8/2) gave a colourless oil (0.042 g, 62%).

^{1}H NMR (250 MHz, $CDCl_3$): δ=7.78 (d, ^3J=8.2 Hz, 2H), 7.36 (d, ^3J=8.2 Hz, 2H), 4.44 (t, ^3J=5.1 Hz, 1H), 3.62 (dq, ^3J=9.3 Hz, $^3J'$=7.1 Hz, 2H), 3.46 (dq, ^3J=9.3 Hz, $^3J'$=7.1 Hz, 2H), 3.36 (t, ^3J=7.5 Hz, 2H), 2.87 (t, ^3J=7.5 Hz, 2H), 2.43 (t+s, 5H), 1.50-1.67 (m, 4H), 1.19 (t, ^3J=7.1 Hz, 6H); ^{13}C NMR (75 MHz, $CDCl_3$): δ=205.9, 145.0, 135.9, 130.0, 128.0, 102.5, 61.2, 50.6, 42.4, 35.1, 32.8, 21.6, 18.8, 15.3; IR (film) ν =2975, 2929, 2896, 1718, 1597, 1447, 1408, 1376, 1315, 1147, 1086, 1059 cm^{-1}; HRMS (ESI): $[M+Na]^+$ Calcd. for $C_{18}H_{28}O_5NaS$: 379.1555. Found 379.1566.

1-(4-Isopropylcyclohex-1-enyl)-5-tosylpentan-3-one (15b)

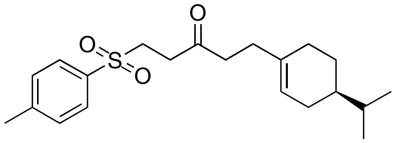

The title compound **15b** was prepared following the general procedure for the radical reductions (method A), starting from xanthate **15a** (0.19 g, 0.39 mmol) and Et_3B (1M solution in hexanes, 1.97 mL, 1.97 mmol, 5 eq.). Silica gel column chromatography (eluent heptane/EtOAc = 9/1) gave a white solid (0.092 g, 64%).

m.p. 89.5-91.0°C; ^{1}H NMR (250 MHz, $CDCl_3$): δ=7.76 (d, ^3J=8.1 Hz, 2H), 7.34 (d, ^3J=8.1 Hz, 2H), 5.3 (bs, 1H), 3.33 (t, ^3J=7.6 Hz, 2H), 2.86 (t, ^3J=7.6 Hz, 2H), 2.50 (t, ^3J=7.6 Hz, 2H), 2.43 (s, 3H), 2.15 (t, ^3J=7.5 Hz, 2H), 2.05-1.58 (m, 5H), 1.50-1.36 (m, 1H), 1.24-1.11 (m, 2H), 0.86 (2d, 6H); ^{13}C

NMR (75 MHz. $CDCl_3$): δ=206.1, 145.0, 136.0, 135.7, 130.0, 128.0, 121.6, 50.6, 41.2, 40.0, 35.0, 32.2, 31.3, 29.1, 28.9, 26.3, 21.6, 19.9, 19.6; IR (neat): ν =2914, 1712, 1311, 1284, 1144, 1085, 819, 656 cm^{-1}; HRMS (ESI): $[M+Na]^+$ Calcd. for $C_{22}H_{30}O_3NaS$: 385.1813. Found: 385.1798.

3-Deoxy-1,2:5,6-di-*O*-isopropylidene-α-D-glucofuranose (20)

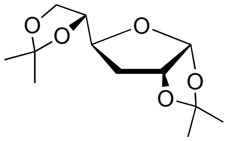

The title compound **20** was prepared following the general procedure for the radical reductions (method B), Et_3B (1M solution in hexanes, 1.0 mL, 1.0 mmol, 5 eq.) in 8 mL $(CH_2Cl)_2$. Xanthate **19** (0.070 g, 0.2 mmol dissolved in $(CH_2Cl)_2$ (1 mL). Silica gel column chromatography (eluent heptane/EtOAc = 9/1 then 8/2) gave a **20** (0.028 g, 57%). [16]

The title compound **20** was also prepared following the general procedure for the radical reductions (method B), Et_3B (1M solution in hexanes, 0.95 mL, 0.95 mmol, 5 eq.) in $(CH_2Cl)_2$ (8 mL). Xanthate **21** (0.070 g, 0.19 mmol) dissolved in $(CH_2Cl)_2$ (1 mL). Silica gel column chromatography gave compound **20** (0.023 g, 50%).

The title compound **20** was also prepared following the general procedure for the radical reductions (method B), Et_3B (1M solution in hexanes, 1.12 mL, 1.12 mmol, 5 eq.) in Et_2O (5 mL). Iodide **22** (0.083 g, 0.224 mmol) dissolved in $(CH_2Cl)_2$ (1 mL). Silica gel column chromatography gave compound **20** (0.023 g, 80%)

^1H NMR (300 MHz, $CDCl_3$): δ=5.82 (d, ^3J=3.7 Hz, 1H), 4.76 (t, ^3J=4.2 Hz, 1H), 4.26-4.06 (m, 3H), 3.86-3.78 (m, 1H), 2.19 (dd, ^3J=3.7 Hz, and $^3J'$=13.5 Hz, 1H), 1.82-1.71 (m, 1H), 1.51 (s, 3H), 1.42 (s, 3H), 1.35 (s, 3H), 1.32 (s, 3H); ^{13}C NMR (75 MHz, $CDCl_3$): δ=111.2, 109.6, 105.5, 80.4, 78.5, 76.7, 67.1, 35.1, 26.7, 26.4, 26.1, 25.1; IR (neat): ν = 1371, 1211, 1162, 1058, 1017, 843 cm^{-1}; HRMS (ESI): $[M+Na]^+$ Calcd. for $C_{12}H_{20}O_{5:}Na$: 267.1208. Found: 267.1223.

Surzur-Tanner/Giese Rearrangement:

1,5-Anhydro-2,3,4,6-tetra-O-acetyl-D-glucitol (17b) and tetra-*O*-acetyl-α-D-*arabino*-2-deoxy-hexopyranose (18)

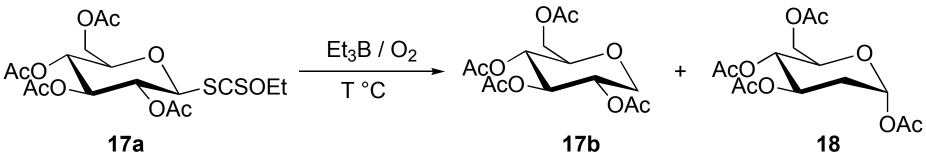

T°C = 20°C: To a mixture of Et_3B (1M solution in hexanes, 0.74 mL, 0.74 mmol, 5 eq.) in $(CH_2Cl)_2$ (5 mL) at 20°C was added a solution of xanthate **17a** (0.067 g, 0.14 mmol) dissolved in $(CH_2Cl)_2$ (0.5 mL) with the aid of a syringe pump over 15 min. Air (20 mL) was added simultaneously with a second syringe pump during 2 h. Silica gel column chromatography (eluent heptane/EtOAc = 9/1 then 8/2) gave a mixture of compounds **17b** and **18** (0.044 g, 89%). The ratio **17b**/**18** (50/50) was determined by ^{1}H NMR.

T°C = -20°C: The same procedure as above was applied, except that the reaction temperature was maintained at -20°C during all the reaction time (2 h). Et_3B (1M solution in hexanes, 0.46 mL, 0.46 mmol, 5 eq.) in $(CH_2Cl)_2$ (5 mL) at -20°C. Xanthate **17a** (0.042 g, 0.093 mmol) was dissolved in $(CH_2Cl)_2$ (0.5 mL). Silica gel column chromatography (eluent heptane/EtOAc = 9/1 then 8/2) gave a mixture of compounds **17b** and **18** (0.028 g, 94%). The ratio **17b**/**18** (> 95/5) was determined by ^{1}H NMR.

T°C = 60°C: The same procedure as above was applied, except that the reaction temperature was maintained at 60°C during all the reaction time (2 h). Et_3B (1M solution in hexanes, 5.9 mL, 5.9 mmol, 5 eq.) in $(CH_2Cl)_2$ (5 mL) in a two-neck round bottom flask equipped with a condenser at 60°C. Xanthate **17a** (0.053 g, 0.12 mmol) dissolved in $(CH_2Cl)_2$ (0.5 mL). Silica gel column chromatography (eluent heptane/EtOAc = 9/1 then 8/2) gave a mixture of compounds **17b** and **18** (0.032 g, 82%). The ratio **17b**/**18** (10/90) was determined by ^{1}H NMR.

Compound **17b**: Literature.[17,18]

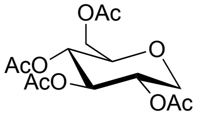

^{1}H NMR (300 MHz, $CDCl_3$): δ=5.21 (t, ^3J=9.5 Hz, 1H), 5.02 (m, 2H), 4.25-4.09 (m, 3H), 3.60 (dq, ^3J=10 Hz, ^3J=2.4 Hz, 1H), 3.31 (t, ^3J=11 Hz, 1H), 2.10 (s, 3H), 2.04 (s, 6H), 2.03 (s, 3H); ^{13}C NMR (75 MHz, $CDCl_3$): δ=170.6, 170.3, 169.7, 169.5, 76.5, 73.7, 68.9, 68.5, 66.9, 62.2, 20.7, 20.6; IR (neat): ν =1731, 1367, 1212, 1193, 1122, 1051, 1012, 970, 922 cm^{-1}; HRMS (ESI): $[M+Na]^+$ Calcd. for $C_{14}H_{20}O_{9:}$Na: 355.1005. Found: 355.1005.

Compound **18**: Literature[19].[19]

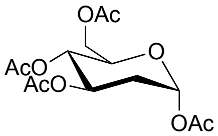

^{1}H NMR (250 MHz, $CDCl_3$): δ=6.27 (bd, ^3J=2.5 Hz, 1H), 5.32 (ddd, ^3J=5.3 Hz, ^3J=1.8 Hz, ^3J=4.3 Hz, 1H), 5.09 (t, ^3J=9.7 Hz, 1H), 4.32 (dd, ^3J=4.7 Hz, ^3J=12.9 Hz, 2H), 4.06 (m, 2H), 1.97 (dd, ^3J=1.9 Hz, ^3J=3.6 Hz, 1H), 2.27 (ddd, ^3J=1.2 Hz, ^3J=5.3 Hz, ^3J=13.5 Hz, 1H), 2.14 (s, 3H), 2.09 (s, 3H), 2.06 (s, 3H), 2.04 (s, 3H); ^{13}C NMR (75 MHz, $CDCl_3$): δ=170.7, 170.2, 169.7, 168.9, 90.9, 70.2, 68.7, 68.5, 61.9, 33.9, 21.0, 20.9, 20.69, 20.65; IR (neat): ν = 1737, 1366, 1210, 1030, 903, 731 cm^{-1}; HRMS (ESI): $[M+Na]^+$ Calcd. for $C_{14}H_{20}O_9$Na: 355.1005. Found: 355.0996.

References:

[1] Perrin, D. D.; Armarego, W. L. F.; Perrin, D.R. Purification of Laboratory Chemicals, Pergamon Press **1980**.

[2] Whitham, G. H.; Bridges, A. J. *J. Chem. Soc. Perkin. Trans.* 1 **1975**, 1603-1606.

[3] Batanero, B.; Picazo, O.; Barba, F. *J. Org. Chem.* **2001**, *66*, 320-322. (b) Boivin, J.; Pothier, J.; Zard, S. Z., *Tetrahedron Lett.* **1999**, *40*, 3701-3704.

[4] Boivin J.; Jrad, R.; Juge, S.; Nguyen, V. T. *Org. Lett.* **2003**, *5*, 1645-1648.

[5] Pinnick, H.W.; Reynolds, M.A. *J. Org. Chem.* **1979**; *44*; 160-161.

[6] Gueyrard, D.; Tatibouët, A.; Gareau, Y.; Rollin P. *Org. Lett.* **1999**, 1, 521-522.

[7] Barton, D. H. R.; Stick, R. V.; Subramanian, R. *J. Chem. Soc. Perkin Trans. 1* **1976**, 19, 2112-2116.

[8] Rasmussen, J. R.; Slinger, C. J.; Kordish, R. J.; Newman-Evans, D. D. *J. Org. Chem.* **1981**, 46, 4843-4846.

[9] Bell, R. H.; Horton, D.; Williams, D. M.; Winter-Mihaly, E. *Carbohydr. Res.* **1977,** 58, 109-124.

[10] Ollivier, C.; Renaud, P. *J. Am. Chem. Soc.* **2001**, 123, 4717-4727.

[11] Cesare, P. D.; Gross, B. *Synthesis* **1980**, 714-715.

[12] Bolon, P.; Jahnke, T. S.; Nair, V. *Tetrahedron* **1995**, 51, 10443-10452.

[13] Vismara, E.; Donna, A.; Minisci, F.; Naggi, A.; Pastori, N. Torri, G. *J. Org. Chem.* **1993**, *58*, 959-963.

[14] Gopalan, A. S.; Jacobs, H. K. *Tetrahedron* Lett. **1990**, *31*, 5575-5578.

[15] (a) Ralston; Christensen *Ind. Eng. Chem.* **1937**, *29*, 194. (b) Gilman; Ford *Iowa State Coll. J. Sci.* **1938/1939**, *13*, 140. (c) Koelbel et al. *Brennst. Chem.* **1959**, *40*, 281, 282-285; *Chem. Abstr.* **1960**, 7521. (d) Patent CIBA Ltd.; NL **1963**; *Chem. Abstr.* **1965**, *62*, 4148a.

[16] Prudhomme, D. R.; Wang, Z.; Rizzo, C. J. *J. Org. Chem.* **1997**, 62, 8257-8260.

[17] Ugi, I.; Meyr, R. *Ber.* **1960**, 93, 247.

[18] Barton, D. H. R.; Bringmann, G.; Lamotte, G.; Motherwell, W. B.; Hay-Motherwell, R. S.; Porter A. E. A. *J. Chem. Soc. Perkin. Trans.* 1 **1980**, 2657-2664.

[19] Yamazaki, O.; Togo, H.; Matsubayashi, S.; Yokoyama, M. *Tetrahedron* **1999**, 55, 3735-3747.
